# Medial preoptic CCKAR mediates anxiety and aggression induced by chronic emotional stress in male mice

**DOI:** 10.1093/nsr/nwaf152

**Published:** 2025-05-21

**Authors:** Meng-Yu Tang, Yan-Yi Zhang, Lin Lin, Lin-Lin Wu, Meng-Ting Hu, Li-Heng Tan, Chen-Xi Yu, Hao Wang, Yan-Qin Yu, Yu Ding, Jia-Xuan Han, Hailan Hu, Xiao-Ming Li, Hong Lian

**Affiliations:** Department of Neurology and Department of Psychiatry, the Second Affiliated Hospital, Zhejiang University School of Medicine, Hangzhou 310058, China; Nanhu Brain-computer Interface Institute, Hangzhou 311100, China; NHC and CAMS Key Laboratory of Medical Neurobiology, MOE Frontier Center of Brain Science and Brain-machine Integration, School of Brain Science and Brain Medicine, Zhejiang University, Hangzhou 310058, China; School of Laboratory Medicine and School of Bioengineering, Hangzhou Medical College, Hangzhou 310053, China; Department of Neurology and Department of Psychiatry, the Second Affiliated Hospital, Zhejiang University School of Medicine, Hangzhou 310058, China; NHC and CAMS Key Laboratory of Medical Neurobiology, MOE Frontier Center of Brain Science and Brain-machine Integration, School of Brain Science and Brain Medicine, Zhejiang University, Hangzhou 310058, China; Department of Neurology and Department of Psychiatry, the Second Affiliated Hospital, Zhejiang University School of Medicine, Hangzhou 310058, China; NHC and CAMS Key Laboratory of Medical Neurobiology, MOE Frontier Center of Brain Science and Brain-machine Integration, School of Brain Science and Brain Medicine, Zhejiang University, Hangzhou 310058, China; NHC and CAMS Key Laboratory of Medical Neurobiology, MOE Frontier Center of Brain Science and Brain-machine Integration, School of Brain Science and Brain Medicine, Zhejiang University, Hangzhou 310058, China; NHC and CAMS Key Laboratory of Medical Neurobiology, MOE Frontier Center of Brain Science and Brain-machine Integration, School of Brain Science and Brain Medicine, Zhejiang University, Hangzhou 310058, China; NHC and CAMS Key Laboratory of Medical Neurobiology, MOE Frontier Center of Brain Science and Brain-machine Integration, School of Brain Science and Brain Medicine, Zhejiang University, Hangzhou 310058, China; NHC and CAMS Key Laboratory of Medical Neurobiology, MOE Frontier Center of Brain Science and Brain-machine Integration, School of Brain Science and Brain Medicine, Zhejiang University, Hangzhou 310058, China; Department of Neurology and Department of Psychiatry, the Second Affiliated Hospital, Zhejiang University School of Medicine, Hangzhou 310058, China; Institute of Pharmacology & Toxicology, College of Pharmaceutical Sciences, Zhejiang University, Hangzhou 310058, China; Affiliated Mental Health Centre and Hangzhou Seventh People's Hospital, Zhejiang University School of Medicine, Hangzhou 310058, China; Lingang Laboratory, Shanghai 200031, China; Department of Neurology and Department of Psychiatry, the Second Affiliated Hospital, Zhejiang University School of Medicine, Hangzhou 310058, China; Nanhu Brain-computer Interface Institute, Hangzhou 311100, China; NHC and CAMS Key Laboratory of Medical Neurobiology, MOE Frontier Center of Brain Science and Brain-machine Integration, School of Brain Science and Brain Medicine, Zhejiang University, Hangzhou 310058, China; NHC and CAMS Key Laboratory of Medical Neurobiology, MOE Frontier Center of Brain Science and Brain-machine Integration, School of Brain Science and Brain Medicine, Zhejiang University, Hangzhou 310058, China; NHC and CAMS Key Laboratory of Medical Neurobiology, MOE Frontier Center of Brain Science and Brain-machine Integration, School of Brain Science and Brain Medicine, Zhejiang University, Hangzhou 310058, China; Affiliated Mental Health Centre and Hangzhou Seventh People's Hospital, Zhejiang University School of Medicine, Hangzhou 310058, China; Department of Neurology and Department of Psychiatry, the Second Affiliated Hospital, Zhejiang University School of Medicine, Hangzhou 310058, China; NHC and CAMS Key Laboratory of Medical Neurobiology, MOE Frontier Center of Brain Science and Brain-machine Integration, School of Brain Science and Brain Medicine, Zhejiang University, Hangzhou 310058, China; Department of Neurology and Department of Psychiatry, the Second Affiliated Hospital, Zhejiang University School of Medicine, Hangzhou 310058, China; Nanhu Brain-computer Interface Institute, Hangzhou 311100, China; Research Center of System Medicine, School of Basic Medical Sciences, Zhejiang University, Hangzhou 310058, China

**Keywords:** medial preoptic area, cholecystokinin A receptor, anxiety, aggression

## Abstract

Anxiety disorders frequently accompany aggression, with their co-occurrence predicting greater functional impairment and poor prognosis. Nevertheless, the underlying neural mechanisms remain elusive, primarily due to a lack of appropriate animal models. Here, we designed a chronic conspecific outsider stress (CCS) model in which male mice underwent perceived social threats and exhibited increased anxiety-like behaviors accompanied by aggression. CCS led to *Fos* activation and hyperexcitability of GABAergic neurons in the medial preoptic area (mPOA). Inhibition of mPOA GABAergic (mPOA^Gad2^) neurons rescued CCS-induced anxiety-like and aggressive behaviors, whereas activating these cells induced susceptibility to CCS. Moreover, CCS upregulated the mRNA and protein expression of the sexual-dimorphic gene, cholecystokinin A receptor (CCKAR)-encoding *Cckar* gene in the mPOA. Importantly, the knock-down and overexpression of CCKAR in the mPOA^Gad2^ neurons had alleviating and promoting effects on anxiety-like and aggressive behaviors, aligning with decreased and increased excitability by the anxiolytic CCKAR antagonist MK-329 and the anxiogenic CCKAR agonist A71623 in mPOA^Gad2^ neurons, respectively. Overall, our study characterizes a novel mouse model of anxiety disorders accompanied by aggression and the neuronal subpopulation and molecular mediator of the aberrant behaviors provide potential targets of intervention for anxiety disorders with aggression.

## INTRODUCTION

Individuals experiencing psychological distress may exhibit internalizing (e.g. anxiety, depression, social avoidance) or externalizing responses (e.g. aggression) [1,2], the dysregulation of which can precipitate a variety of mental disorders. Anxiety disorders represent a significant proportion of internalizing disorders, with a lifetime prevalence rate of ≤33.7% [3]. Despite the growing body of research delineating the connections between anxiety disorders and other forms of internalizing behaviors (e.g. social avoidance) [4,5], explorations into the link between anxiety disorders and externalizing behaviors remain scarce. Epidemiological studies suggest that patients who are diagnosed with anxiety disorders are significantly more likely to exhibit aggressive behaviors than individuals without mental illness [6], with their co-occurrence predicting greater functional impairment and poorer prognosis, thus imposing a severe disease burden on the affected individual, their families and broader society [7,8]. In addition, neuroimaging and animal research on anxiety and aggression have identified some commonalities in the brain regions and neurochemical systems involved [9,10]. However, the precise neural mechanisms that drive this co-occurrence have yet to be fully elucidated. While studies have demonstrated the efficacy of social-isolation stress in triggering anxiety and aggressive behaviors [11–13], the integration of social contact and social interaction—the essential elements of human daily life—remains largely absent in models of co-occurring anxiety and aggression.

To address this challenge, we developed a chronic conspecific stress (CCS) model that concurrently induces anxiety and aggression by subjecting a male mouse, paired with a female, to continual threats from an outsider male conspecific. Through the application of the CCS model, we elucidated the molecular mechanisms underlying the simultaneous manifestation of anxiety-like and aggressive behaviors. Consequently, the CCS model has emerged as a straightforward and efficient approach for the study of co-occurring anxiety and aggression, with the identification of neural mechanisms offering promising novel therapeutic targets for anxiety disorders.

## RESULTS

### CCS induces anxiety-like and aggressive behaviors in male mice

**Figure 1.**
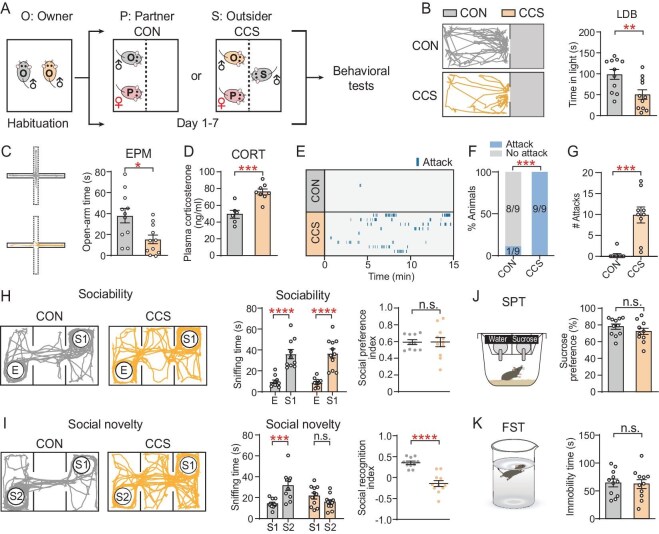
Seven-day CCS exposure induces anxiety-like and aggressive behaviors in CCS male owner mice. (A) Experimental design of CCS paradigm and timeline of behavioral tests. CON, control; CCS, chronic conspecific outsider stress. (B) Representative trajectories (left) and quantification (right) of time spent in light chamber in the LDB. (C) Representative trajectories (left) and quantification (right) of time spent in the open arms in the EPM. (D) Quantification of plasma corticosterone levels. (E) Representative raster plots of nine mice from each group showing attacks launched by CON and CCS male owners. (F) Percentage of CON and CCS mice showing attacks. (G) Total number of outsider-directed attacks launched by CCS mice. (H) Representative trajectories (left) and quantification of sniffing time and social preference index (right). (I) Representative trajectories (left) and quantification of sniffing time and social recognition index (right). (J) Sucrose preference in SPT. (K) Total immobility time in FST. Values are means ± SEM. except (F). *P < 0.05; **P < 0.01; ***P < 0.001; ****P < 0.0001; n.s., no significance (see Table S1 for statistics and *n* numbers). See also Supplementary Videos S1 and S2.

In the CCS paradigm, a male owner mouse (O) was housed with a female partner (P) on one side of a cage and an outsider mouse (S) was housed on the other side of the cage, separated by a transparent perforated partition. In contrast, control mice (CON) referred to female-paired owner males that were not exposed to outsiders. Owners in the CON and CCS groups were then subjected to behavioral tests following the 7-day exposure period to evaluate their levels of anxiety and aggression (Fig. 1A). To assess anxiety-related behaviors, two approach-avoidance conflict tests were used: the light/dark box test (LDB) and the elevated plus-maze test (EPM) [5,14,15]. The CCS mice spent less time in the light chamber in the LDB (Fig. 1B) and in the open arms in the EPM (Fig. 1C), with a consistent increase in plasma corticosterone levels (CORT, Fig. 1D). Notably, upon removal of the partition, all CCS mice (9/9) initiated aggressive encounters with the outsider mice, in contrast to the CON mice (1/9) (Supplementary Videos S1 and S2, and Fig. 1E and F). Additionally, the CCS mice engaged in a greater number of attacks than the CON mice (Fig. 1G), indicating enhanced aggression in the CCS mice. Given that aggression may represent a partial manifestation of social dysfunctions in CCS mice, the three-chamber social preference test (TCT) was conducted to further assess sociability and social novelty. The CCS mice spent more time around a stranger mouse (Stranger 1, S1) than that around an empty cup and displayed a similar social preference index to CON mice (Fig. 1H). However, CCS mice showed no preference for a newly introduced stranger mouse (Stranger 2, S2) over S1 and thus had a significantly decreased social recognition index (Fig. 1I), indicating impaired social recognition. The results of the sucrose-preference test (SPT) (a measurement of anhedonia) and the forced swimming test (FST) (a measurement of behavioral despair) demonstrated that CCS did not induce depression-like behaviors (Fig. 1J and K). Collectively, the CCS mice exhibited patterns of anxiety-like behaviors, increased aggression and social recognition impairment.

To evaluate the sex specificity of the CCS paradigm, the behaviors of male partner-paired female owners facing female or male outsiders were measured. Female mice that were subjected to these modified CCS paradigms did not exhibit anxiety-like behaviors or social recognition impairment (Fig. S1). No females showed aggressive behavior, consistently with previous studies which showed that female mice rarely show aggression unless they are lactating or parental [16–19], which is probably attributed to weaker possessiveness, awareness of territory, mates and social-bonds preservation compared with male mice. These results indicate that the behavioral responsiveness to the CCS model is male-specific.

To identify the sex configurations necessary for the partner and outsider mice, the standard CCS paradigm was modified by replacing the female partner with a male partner or changing the sex of the outsider mouse from male to female (Fig. S2A). Observations revealed that no male owner mice exhibited anxiety-like behaviors, aggression or social recognition impairment (Fig. S2B–E). Another finding indicated that the male outsider must pose a sufficient threat to the male owner to elicit the above behaviors, with less intimidating objects/mice, such as toys or juvenile males, failing to impact anxiety-like behaviors or social recognition impairment in owner mice (Fig. S2F–J). Thus, the presence of a male owner, a female partner and a competitive male outsider appears to be an essential requirement to induce anxiety-like behaviors, aggression and impaired social recognition within the CCS framework.

Furthermore, we investigated how CCS mice perceived threats from outsiders. Mice received a bilateral ZnSO4 irrigation (20 μl, 2%) of the nasal cavity to induce olfactory dysfunction and then underwent the CCS paradigm. Compared with CCS mice with normal olfaction (received saline irrigation), anxiety-like behavior and social impairment were rescued in CCS mice with impaired olfaction (Fig. S3A–G). To test whether acoustic interactions had contributed to the behavioral changes, we experimentally induced hearing loss in mice by using ototoxin injection (1000 mg/kg of kanamycin followed 30–45 min later by a single dose of 400 mg/kg of furosemide) [20]. Auditory lesion did not improve the behavioral abnormalities induced by CCS, as ototoxin-treated CCS mice (O-CCS) displayed a similar level of anxiety and aggression to saline-treated CCS mice (S-CCS) (Fig. S3H–N). Additionally, a gray opaque partition was used to block the visibility of outsiders. Similarly to the transparent partition, the opaque partition did not affect the induction of anxiety-like and aggressive behaviors by the CCS paradigm (Fig. S3O-U). Therefore, olfactory inputs are required for CCS mice to perceive threats from outsiders.

### mPOA GABAergic neurons bidirectionally regulate anxiety-like and aggressive behaviors

Given the male specificity of behavioral responses to the CCS paradigm (Fig. 1 and Fig. S1), we investigated whether CCS induced behavioral changes through sexually dimorphic brain regions. CON and CCS mice were sacrificed 30 min after partition withdrawal (which enhanced the outsider exposure) and *Fos* RNAscope staining was performed to compare the activity changes of four sexually dimorphic brain regions [21]: the medial preoptic area (mPOA), the bed nucleus of stria terminalis (BNST), the medial amygdala (MeA) and the ventromedial hypothalamus (VMH). CCS mice exhibited a significant increase in the number of *Fos*-positive (*Fos^+^*) cells in the mPOA compared with CON mice, which was not observed in the BNST, MeA or VMH (Fig. 2A and B). The mPOA primarily consists of GABAergic and glutamatergic neurons, and plays pivotal roles in mating, aggression and affiliative social behaviors [22–24]. Through colocalization analysis of *Fos* and neuronal subtype marker genes (*Gad2* for GABAergic and *Slc17a6* for glutamatergic neurons), an increase in GABAergic (Fig. 2C and D) but not glutamatergic (Fig. S4A and B) *Fos^+^* neurons was observed in CCS mice. Moreover, voltage patch-clamp recordings of mPOA GABAergic (mPOA^Gad2^) neurons, labeled by adeno-associated viral (AAV)-mediated green fluorescent protein (GFP) expression under a GAD65 promoter whose specificity had been verified by co-staining with *Gad2* (71%) and *Slc17a6* (29%) (Fig. 2E and Fig. S4C and D), revealed a significant increase in the neuronal excitability in the CCS mice, including more depolarized resting membrane potential (RMP, Fig. 2F) and decreased rheobase current (Fig. 2G). No significant differences were noted in the input resistance (*R*_in_, Fig. S4E) or action potential (AP)-related parameters, including the amplitude (Fig. S4F), half-width (Fig. S4G) and after-hyperpolarization potential (AHP) amplitude (Fig. S4H). These results suggest that exposure to CCS leads to the hyperexcitability of mPOA^Gad2^ neurons.

**Figure:**
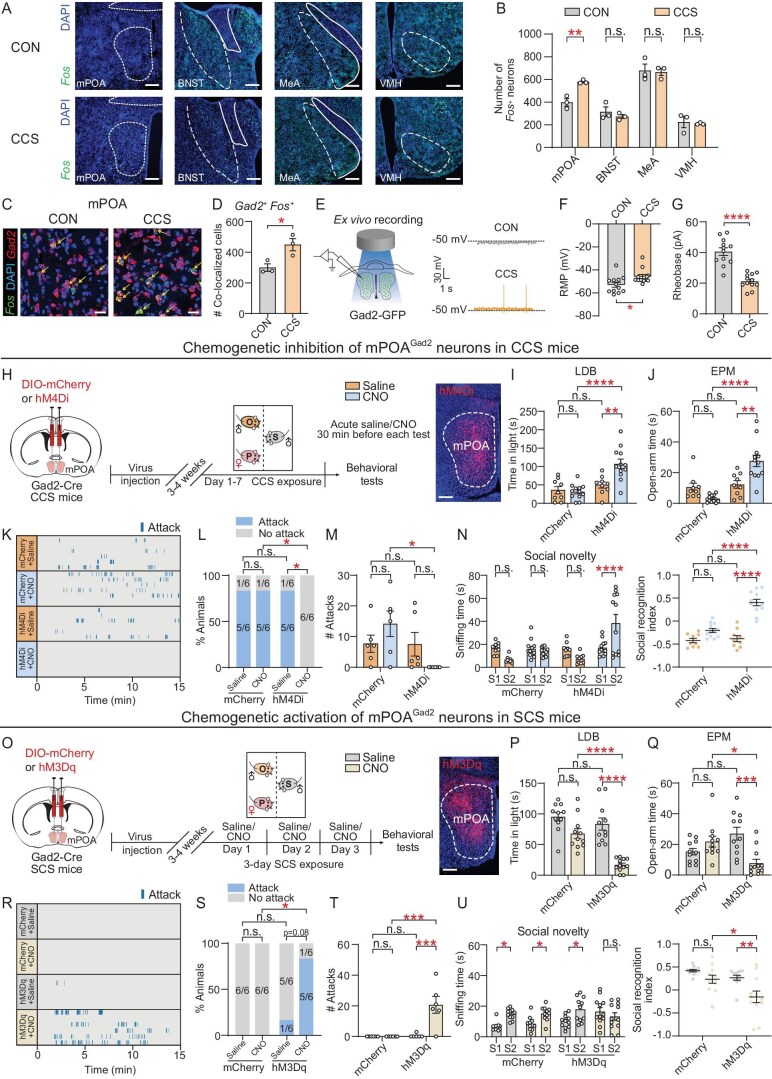
CCS activates mPOA^Gad2^ neurons that bidirectionally regulate anxiety-like and aggressive behaviors. (A) Representative images and (B) quantification of *Fos^+^* cells in the mPOA, BNST, MeA and VMH in CON and CCS mice. Scale bar, 200 μm. (C) Representative images and (D) quantification of cells co-expressing *Gad2* and *Fos* in the mPOA. Arrows indicate *Fos^+^* neurons expressing *Gad2.* Scale bar, 20 μm. (E) Left, experimental set-up for *ex vivo* electrophysiology. Right, representative RMP traces of mPOA^Gad2^ neurons in CON and CCS male mice. Dotted line refers to the voltage of −50 mV. (F) RMP and (G) rheobase of mPOA^Gad2^ cells in CON and CCS mice. (H and O) Left, experimental procedure for testing behaviors of (H) CCS mice with chemogenetic inhibition in mPOA^Gad2^ neurons or (O) mice subjected to 3-day SCS paradigm with chemogenetic activation of mPOA^Gad2^ neurons. Right, representative fluorescence image showing viral injection in the mPOA. Scale bar, 200 μm. (I and P) Quantification of time spent in light chamber in the LDB by viral-infected mice treated with saline or CNO. (J and Q) Quantification of time spent in open arms in the EPM. (K and R) Raster plots showing outsider-directed attacks. (L and S) Percentage of CCS or SCS mice showing attacks. (M and T) Total number of outsider-directed attacks launched by CCS or SCS mice. (N and U) Quantification of sniffing time for social novelty and social recognition index in TCT. Values are means ± SEM. except (L) and (S). *P < 0.05; **P < 0.01; ***P < 0.001; ****P < 0.0001; n.s., no significance (see Table S1 for statistics and *n* numbers).

To investigate whether acute mPOA^Gad2^ neuron inhibition can block CCS-induced anxiety-like and aggressive behaviors, AAVs expressing Cre-dependent (DIO) hM4Di (human M4 muscarinic receptor coupled to Gi) were bilaterally injected into the mPOA of Gad2-Cre mice. Clozapine-N-oxide (CNO, 5 μM) incubation markedly decreased the firing rate of hM4Di-expressing mPOA^Gad2^ neurons at –40 mV, a more depolarized potential (Fig. S4I). Upon acute intraperitoneal (i.p.) administration of CNO (1 mg/kg) treatment 30 min before each test (Fig. 2H), the hM4Di-expressing CCS mice spent more time in the light chamber and open arms in the LDB and EPM, respectively (Fig. 2I and J). Additionally, chemogenetic inhibition of the mPOA^Gad2^ neurons led to a substantial reduction in both the probability and frequency of aggressive attacks (Fig. 2K–M) and ameliorated social recognition impairment (Fig. 2N). Considering the kinetic, metabolic as well as potential off-target effects of CNO, deschloroclozapine (DCZ, 0.1 mg/kg)—a new potent and selective chemogenetic actuator [25]—was applied to repeat the above experiments (Fig. S4K). In the hM4Di-expressing CCS mice, DCZ had the same behavioral effects as CNO (Fig. S4L–Q). As control, the chemogenetic inhibition of mPOA^Gad2^ neurons in naïve mice that were not exposed to CCS did not generate a significant effect on anxiety-like and aggressive behaviors (Fig. S4R–V). As some excitatory neurons may also express *Gad2*, leading to mixed effects from both excitatory and inhibitory neurons during the manipulation of mPOA^Gad2^ neurons, we conducted electrophysiological recordings and chemogenetic inhibition of mPOA excitatory neurons in Vglut2-Cre mice. The electrophysiological properties of mPOA glutamatergic (mPOA^Vglut2^) neurons, marked by viral Cre-dependent GFP expression in Vglut2-Cre CCS male mice, did not show any significant differences between CON and CCS mice (Fig. S5A–G). Also, chemogenetic inhibition of mPOA^Vglut2^ neurons did not obviously affect behavioral performance in CCS mice (Fig. S5H–N). Thus, these results support the role of mPOA GABAergic neurons in the manifestation of behavioral abnormalities induced by CCS exposure.

Next, to determine whether increased mPOA^Gad2^ neuronal activity is sufficient for replicating CCS-induced behavioral abnormalities, a 3-day subthreshold CCS (SCS) paradigm that did not elicit anxiety-like or aggressive behaviors in male owners was applied (Fig. S6). Gad2-Cre mice selectively expressing Cre-dependent mCherry or hM3Dq (the human M3 muscarinic receptor coupled to Gq) in the mPOA received daily i.p. injections of saline or CNO (0.5 mg/kg) during the SCS paradigm (Fig. 2O). CNO (5 μM) incubation significantly increased the firing rate of hM3Dq-expressing mPOA^Gad2^ neurons at –70 mV, a more hyperpolarized potential (Fig. S4J). Compared with CON mice, SCS mice with activated mPOA^Gad2^ neurons spent significantly less time exploring the light chamber in the LDB (Fig. 2P) and open arms in the EPM (Fig. 2Q) and exhibited an increase in aggressive behaviors (Fig. 2R–T) and social recognition impairment (Fig. 2U). Collectively, these results indicate that mPOA^Gad2^ neurons bidirectionally regulate anxiety-like and aggressive behaviors induced by CCS.

### Loss of function of CCKAR in the mPOA^Gad2^ reverses anxiety-like and aggressive behaviors in CCS mice

**Figure 3.**
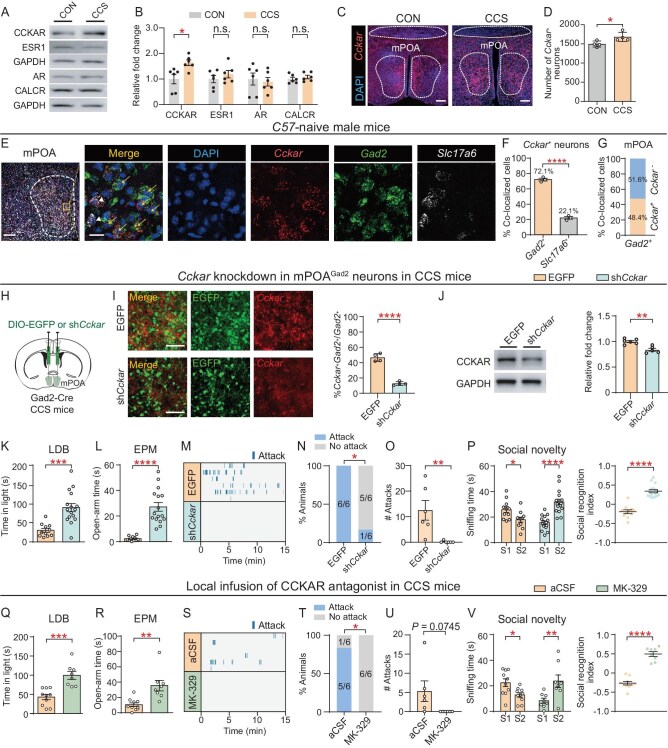
CCKAR loss of function in the mPOA^Gad2^ neurons reverses CCS-induced anxiety-like and aggressive behaviors. (A) Representative blot image and (B) quantification of CCKAR, ESR1, AR, CALCR and GAPDH levels in the mPOA of CON and CCS mice. Protein expression was normalized by the control level. (C) Representative RNAscope images and (D) quantification of *Cckar* expression in mPOA of CON and CCS mice. Scale bar, 200 μm. (E) Representative RNAscope images showing *Cckar, Gad2* and *Slc17a6* expression in the mPOA of *C57*-naïve male mice (left, scale bar, 200 μm) and magnified pictures of the selected region in the square (right, scale bar, 20 μm). Arrows indicate *Cckar^+^* neurons expressing *Gad2*; Arrowheads indicate *Cckar*^+^ neurons expressing *Slc17a6*. (F) Percentage of *Cckar*^+^ neurons expressing *Gad2* or *Slc17a6*. (G) Proportion of *Cckar*^+^ cells in mPOA^Gad2^ neurons. (H) Schematic of viral injection for conditional knock-down of *Cckar* in mPOA^Gad2^ neurons. (I) Left, representative fluorescence images showing expression of viral EGFP protein and *Cckar* mRNA in the mPOA of Gad2-Cre mice injected with Cre-dependent control (EGFP) or knock-down (sh*Cckar*) virus. Scale bar, 100 μm. Right, percentage of *Cckar*^+^ neurons in mPOA^Gad2^ neurons. (J) Representative blots (left) and quantification (right) of CCKAR in the mPOA of AAV-infected mice. (K and Q) Quantification of time spent in light chamber in the LDB by (K) viral-infected or (Q) antagonist-treated CCS mice. (L and R) Quantification of time spent in open arms in the EPM. (M and S) Raster plots showing outsider-directed attacks. (N and T) Percentage of male mice showing attacks. (O and U) Total number of outsider-directed attacks. (P and V) Quantification of sniffing time for social novelty and social recognition index in TCT. Values are means ± SEM. except (N) and (T). *P < 0.05; **P < 0.01; ***P < 0.001; ****P < 0.0001; n.s., no significance (see Table S1 for statistics and *n* numbers).

To elucidate the underlying molecular mechanisms mediating the elevated activity of mPOA^Gad2^ neurons in CCS male mice, we examined the expression of sexually dimorphic genes that have been associated with anxiety or social behaviors in mPOA protein lysates: cholecystokinin A receptor (CCKAR), estrogen receptor 1 (ESR1), androgen receptor (AR) and calcitonin receptor (CALCR) [21,22,24,26]. Western blot analysis indicated that CCKAR, but not the other proteins, was upregulated in the mPOA of CCS mice (Fig. 3A and B). Encoded by the male-upregulated gene *Cckar*, CCKAR is a class-A 7-fold transmembrane G protein-coupled receptors, richly expressed in the cerebral cortex, hippocampus, hypothalamus and other regions [27,28]. Although studies have implicated CCKAR in spatial memory, food intake and olfactory recognition [29–31], whether CCKAR plays a role in anxiety and aggression remains unclear.

Similarly to *Fos*, RNAscope staining of *Cckar* showed that the number of *Cckar-*positive (*Cckar*^+^) neurons was significantly increased in the mPOA (Fig. 3C and D) but not BNST, MeA or VMH of CCS mice (Fig. S7A and B). We performed triple *in situ* hybridization of *Cckar, Gad2* and *Slc17a6* to characterize the sex-dependent cell-type-specific expression pattern of *Cckar* in the mPOA of both sexes. *Cckar* mRNA expression in the mPOA was higher in male than female mice, as reported [21] (Fig. S7C). Further analysis revealed that, in the male mPOA, 72.1% of *Cckar^+^* cells expressed *Gad2* with only 22.1% expressing *Slc17a6* (Fig. 3E and F) and 48.4% of *Gad2*-positive (*Gad2*^+^) neurons expressed *Cckar* (Fig. 3G). In the female mPOA, 53% of *Cckar^+^* cells expressed *Gad2*, while only 18% of *Gad2*^+^ neurons expressed *Cckar* (Fig. S7D–F). Thus, these findings suggest that *Cckar* exhibits preferential expression in male mPOA^Gad2^ neurons and is upregulated in CCS mice.

We next addressed whether the altered *Cckar* expression in mPOA^Gad2^ neurons contributed to CCS-induced behavioral abnormalities. *Cckar* was conditionally knocked down in mPOA^Gad2^ neurons by injecting AAVs expressing Cre-dependent *Cckar*-targeting short-hairpin RNA (sh*Cckar*) into the mPOA of Gad2-Cre mice (Fig. 3H). Control mice were injected with AAV expressing enhanced green fluorescent protein (EGFP) with a scrambled shRNA sequence. Quantification of *Cckar* RNAscope staining and CCKAR immunoblotting confirmed decreased *Cckar* mRNA and CCKAR protein expression in the mPOA of infected mice by viral-mediated knock-down (Fig. 3I and J). In the CCS male mice, *Cckar* knock-down in mPOA^Gad2^ neurons markedly rescued anxiety-like behaviors in the LDB and EPM (Fig. 3K and L), decreased the probability and frequency of attacks (Fig. 3M–O) and restored social novelty (Fig. 3P). Thus, these observations indicate that *Cckar* expression in the mPOA^Gad2^ neurons is required for CCS-induced anxiety-like and aggressive behaviors. As *Cckar* mRNA is also male-enriched in the BNST and MeA [21], we conditionally knocked down *Cckar* in GABAergic neurons of the BNST and MeA in Gad2-Cre male mice and observed no effects on reversing the behavioral abnormalities in CCS mice (Fig. S8). These findings further suggest that *Cckar* in mPOA^Gad2^ neurons acts as a regulator of CCS-induced anxiety-like and aggressive behaviors.

To determine whether *Cckar* in the mPOA is a general regulator of anxiety-like behaviors, a 7-day subthreshold social defeat stress (SSDS) model was developed, based on our previous research [32], to explore the impact of mPOA *Cckar* knock-down on SSDS-induced anxiety (Fig. S9A). In this model, a male mouse was placed into the home cage of an aggressive CD1 mouse for 5 min daily, during which it experienced physical defeat, followed by 24 h of non-physical sensory contact with CD1. CON mice, with mPOA^Gad2^ neurons infected by control AAV, were housed with *C57*-naïve mice in the same two-chamber cage, but were not subjected to any physical or sensory contact with CD1 mice. Compared with the CON mice, the SSDS mice, infected by either control or knock-down AAV, spent markedly less time in the light box in the LDB and open arms in the EPM (Fig. S9B and C), but did not present sociability deficits or depression-like behaviors (Fig. S9D–G). Thus, *Cckar* knock-down in the mPOA^Gad2^ neurons did not impact SSDS-induced behavioral abnormalities. These results together suggest that *Cckar* in mPOA^Gad2^ cells may specifically regulate CCS-induced co-occurring anxiety-like behavior, aggression and social recognition impairment.

To establish a direct link between CCKAR protein and the manifestation of anxiety-like and aggressive behaviors, the activity of CCKAR in CCS mice was pharmacologically manipulated. As the CCS mice overexpressed CCKAR in the mPOA, acute administration of selective CCKAR antagonist MK-329 (also called devazepide) [33] (25 ng in 300 nl per side) into the mPOA of cannula-implanted CCS mice was performed. MK-329 significantly mitigated anxiety-like behaviors, reduced the likelihood of attacks and improved social recognition deficits, in contrast to the vehicle control artificial cerebrospinal fluid (aCSF) (Fig. 3Q–V). These findings suggest that CCKAR hyperactivity in the mPOA underlies behavioral abnormalities induced by CCS.

### Gain of function of CCKAR in mPOA^Gad2^ induces anxiety-like and aggressive behaviors in SCS mice

**Figure 4.**
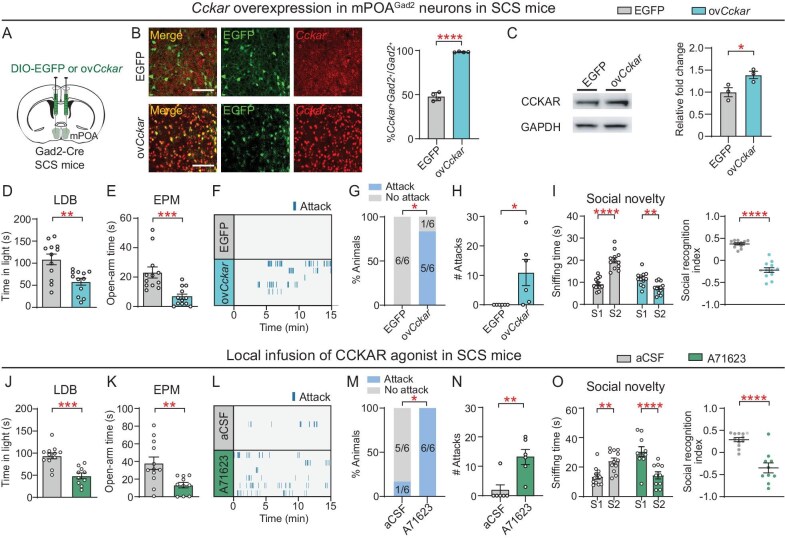
Gain of function of CCKAR in the mPOA^Gad2^ promotes anxiety-like and aggressive behaviors. (A) Schematic of viral injection for conditional overexpression of *Cckar* in mPOA^Gad2^ neurons. (B) Left, representative fluorescence images showing expression of viral EGFP protein and *Cckar* mRNA in the mPOA of Gad2-Cre mice injected with Cre-dependent control (EGFP) or overexpression (ov*Cckar*) virus. Scale bar, 100 μm. Right, percentage of *Cckar*^+^ neurons in mPOA^Gad2^ neurons. (C) Representative blots (left) and quantification (right) of CCKAR in the mPOA lysates of viral-infected mice. (D and J) Quantification of time spent in light chamber in the LDB by (D) viral-infected or (J) agonist-treated SCS mice. (E and K) Quantification of time spent in open arms in the EPM. (F and L) Raster plots showing outsider-directed attacks. (G and M) Percentage of male mice showing attacks. (H and N) Total number of outsider-directed attacks. (I and O) Quantification of sniffing time for social novelty and social recognition index in TCT. Values are means ± SEM. except (G) and (M). *P < 0.05; **P < 0.01; ***P < 0.001; ****P < 0.0001; n.s., no significance (see Table S1 for statistics and *n* numbers).

To evaluate the sufficiency of elevated *Cckar* expression in promoting CCS-induced anxiety-like and aggressive behaviors, *Cckar* was overexpressed in mPOA^Gad2^ neurons through the injection of a Cre-dependent *Cckar*-3xFLAG-P2A-EGFP virus (ov*Cckar*) into the mPOA of Gad2-Cre mice (Fig. 4A). CON mice received an injection of a control AAV expressing only the reporter EGFP. RNAscope staining and immunoblotting confirmed *Cckar* mRNA and CCKAR protein overexpression (Fig. 4B and C). In mice subjected to 3-day SCS, overexpression of *Cckar* in mPOA^Gad2^ neurons markedly increased anxiety-like behaviors in the LDB and EPM (Fig. 4D and E), as well as the probability and frequency of attacks (Fig. 4F–H) and social recognition impairment (Fig. 4I). In addition, pharmacological activation of CCKAR in the mPOA of mice exposed to 3-day SCS through daily infusion of the selective CCKAR agonist A71623 (75 ng in 300 nl per side) [34] induced anxiety-like behaviors, aggression and social recognition impairment (Fig. 4J–O), mirroring the outcomes observed with intra-mPOA CCKAR overexpression and chemogenetic activation of mPOA^Gad2^ neurons. Collectively, the findings from both CCKAR loss- and gain-of-function experiments indicate that CCKAR activity in the mPOA is necessary and susceptible for the exhibition of CCS-induced co-occurring anxiety-like and aggressive behaviors.

### CCKAR regulates neuronal excitability of mPOA^Gad2^ cells

**Figure 5.**
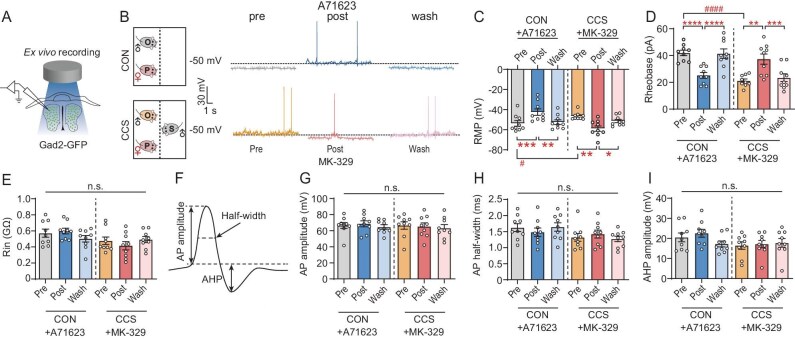
CCKAR regulates excitability of mPOA^Gad2^ neurons. (A) Experimental set-up for *ex vivo* electrophysiology. (B) Representative RMP traces of mPOA^Gad2^ neurons in CON and CCS male mice at stages of pre-/post-incubation and washout of CCKAR antagonist (MK-329) or agonist (A71623). Dotted line represents voltage of −50 mV. (C–E) Quantification of (C) RMP, (D) rheobase and (E) input resistance of labeled mPOA^Gad2^ cells. (F–I) Representative AP trace (F) and quantification of AP amplitude (G), AP half-width (H) and AHP amplitude (I) of labeled mPOA^Gad2^ neurons. Values are means ± SEM. *P < 0.05; **P < 0.01; ***P < 0.001; ****P < 0.0001; n.s., no significance; ^#^*P* < 0.05; ^####^*P* < 0.0001 (see Table S1 for statistics and *n* numbers).

Given that reducing mPOA^Gad2^ neuron excitability and CCKAR activity in the mPOA rescued anxiety-like and aggressive behaviors induced by CCS, while increased excitability and CCKAR activity promoted these behaviors, we hypothesized that CCKAR may modulate CCS-induced behavioral deficits by influencing the excitability of mPOA^Gad2^ neurons. Voltage patch-clamp recordings (Fig. 5A) revealed that perfusion of the CCKAR agonist A71623 in brain slices of CON mice led to higher excitability of mPOA^Gad2^ neurons, as evidenced by depolarized RMP and decreased rheobase, whereas perfusion of the CCKAR antagonist MK-329 largely reversed the hyperexcitability of mPOA^Gad2^ cells in CCS mice (Fig. 5B–D). Treatment with neither A71623 nor MK-329 affected the input resistance (Fig. 5E) or AP-related parameters (Fig. 5F), including the AP amplitude (Fig. 5G), AP half-width (Fig. 5H) and AHP amplitude (Fig. 5I), of mPOA^Gad2^ neurons. As CCKAR is also expressed in some excitatory neurons (Fig. 3F), CCKAR antagonists and agonists might also affect excitatory neuron activity. To test this possibility, we recorded mPOA^Vglut2^ cells labeled by GFP in acute brain slices of Vglut2-Cre mice. Enhancing or suppressing CCKAR did not affect the passive membrane characteristics and AP-related parameters of mPOA^Vglut2^ cells (Fig. S10A–F).

In conclusion, these results imply that CCKAR acts as a molecular switch for CCS-induced concurrent anxiety-like and aggressive behaviors by modifying the excitability of mPOA GABAergic neurons.

## DISCUSSION

In this study, we established a novel CCS mouse model of anxiety accompanied by aggression. At the cellular level, mPOA^Gad2^ regulates CCS-induced behavioral changes whereas, at the molecular level, CCKAR in mPOA^Gad2^ neurons mediate these behaviors by modulating neuronal excitability.

Existing rodent models of anxiety in conjunction with aggression, including acute intervention, transgenic mice and selective breeding models [35–37], fail to incorporate the impact of environmental stress on the induction of aggressive behavior. Early-life stress—a shared risk factor for the emergence of both anxiety disorders and heightened aggression in later life—represents a developmental model for studying the mechanisms underlying concurrent changes in anxiety and aggression [38,39]. However, given that early-life stress can start from infancy, the impact of environmental factors on central-nervous-system development cannot be ignored, introducing considerable limitations to the model. In contrast to the models mentioned above, the novel CCS model adopts adult mice, with the 7 days of CCS exposure sufficient to elicit a stable behavioral phenotype. This approach marks a significant advancement in developing more easy-to-operate animal models for anxiety disorders co-occurring with aggression in adults.

The CON male mice paired with females in the absence of exposure to a conspecific male are not aggressive toward newly introduced stranger males after partition withdrawal. This result seems to be against previous works showing that sexual experience greatly enhances aggressiveness in male mice; however, the discrepancy may have derived from the variance in experimental set-ups and timings of attack recording [40–43]. For instance, Remedios *et al.* reported that a 30-min priming with females promoted aggressiveness of the resident males to attack intruder males in the resident-intruder (RI) tests performed the next day [42]. In our set-up, the CON male mice were always group-housed with female mice and attack recording was performed in the presence of both females and intruders. The lack of aggressiveness in CON mice may have been caused by their preference for social novelty over attack, as they were naïve to non-littermate conspecific intruder males when recording started. Interestingly, Remedios *et al.* described that single-housed male mice subjected to a 3-day RI test paradigm showed little attack but sniffing on Day 1 [42], implicating temporal dynamic neural mechanisms underlying aggression. Whether the neural substrate for aggression in single-housed male mice overlaps with that in CCS mice will require further investigation.

The CCS paradigm utilized a two-chamber cage to prevent physical contact, thereby effectively replicating psychological stressors known to provoke anxiety and aggressive behaviors in humans. In contrast, the social defeat stress involves both physical and emotional stress. While our results showed *Fos* activation in mPOA^Gad2^ neurons by CCS, repeated social defeat stress fails to increase c-fos expression in the mPOA in mice [44,45]. Besides, *Cckar* knock-down, which ameliorates CCS-induced anxiety, did not rescue SSDS-induced anxiety (Fig. S9). Thus, GABAergic neurons and CCKAR in the mPOA may exert stressor-dependent effects on anxiety and aggression.

Our results showed that inhibiting mPOA^Gad2^ cells reduced anxiety-like behaviors and aggression in CCS mice whereas activating these cells in SCS mice exerted opposite effects. The data are opposite to conclusions from recent publications showing that activation of mPOA *Vgat*^+^ GABAergic cells is anxiolytic [46–48] and that inhibiting mPOA *Esr1*^+^ cells (which are mainly GABAergic) increases aggression [49]. The conflicts may have derived from the variance in the GABAergic neuronal subpopulations targeted in different studies. Although the vesicular GABA transporter (vGAT, encoded by *Slc32a1*) and glutamate decarboxylase 2 (GAD65, encoded by *Gad2*) are two predominant markers for GABAergic neurons, *Gad2^+^* neurons may corelease GABA and glutamate whereas virtually all *Slc32a1^+^* neurons synaptically release GABA [50–52]. RNAscope analysis showed that ∼13% of *Gad2^+^* cells in the male mPOA expressed *Slc17a6* (Fig. S11A and B, related to Fig. 3E). However, we demonstrated that the excitability of mPOA^Vglut2^ neurons was unaffected by CCS exposure (Fig. S5A–G), chemogenetic inhibition of mPOA^Vglut2^ neurons did not alter behavioral performance in CCS mice (Fig. S5H–N) and CCKAR antagonists and agonists did not modulate mPOA^Vglut2^ neural activity in acute brain slices (Fig. S10). In addition, RNAscope analysis indicated that *Esr1* and *Cckar* each colocalized with half of the mPOA^Gad2^ neurons and the two subtype neurons overlapped by ∼50% (Fig. S11C–E). Altogether, activating mPOA GABAergic neurons may produce different behavioral outcomes through opto/chemogenetic manipulations when using Gad2-Cre, Vgat-Cre or Esr1-Cre mice. Despite the anxiolytic effect from the activation of mPOA GABAergic neurons using Vgat-Cre-naïve mice in an recent report [46], our work revealed an anxiogenic effect from the activation of mPOA GABAergic neurons in Gad2-Cre SCS mice and an anxiolytic effect from their inhibition in CCS but not naïve male mice (Fig. 2 and Fig. S4K–V). Thus, the complexity introduced by different behavioral paradigms may also serve as a factor that contributes to the paradox.

Olfactory deprivation, rather than auditory or visual deprivation, ameliorated the CCS-induced anxiety and deficits in social recognition (Fig. S3). Notably, MK-329, a selective antagonist of CCKAR, has been previously reported to impair olfactory recognition [29,53], while our findings demonstrate its ability to rescue CCS-induced anxiety (Fig. 3S–V). This suggests that a potential neural circuit conveying olfactory information to the mPOA may be involved in promoting anxiety-like and aggressive behaviors, subject to CCKAR regulation. Indeed, the mPOA receives direct olfactory projections from the accessory olfactory bulb [54] and indirect projections from the MeA and BNST [55]. Notably, these brain regions contain CCK-positive neurons [56,57], which are capable of releasing CCK neurotransmitters to interact with CCKAR in the mPOA. Intriguingly, a recent study revealed that chemosensory input-receiving BNST^Tac1^ neurons release substance P (encoded by *Tac1*) and induce excitatory long-term potentiation in mPOA neurons through the receptor Tacr1 to initiate mating [58], highlighting the modulatory functions of neuropeptides from upstream nuclei in the mPOA on neuronal/synaptic excitability. Moreover, the mPOA strongly projects to the BNST, ventral tegmental area and nucleus accumbens, which regulate anxiety-like behaviors [59–61], as well as to the lateral hypothalamus, lateral septum and periaqueductal gray, known to regulate aggressive behaviors [62–64]. However, further in-depth studies are necessary to reveal the distinct neural circuits underlying co-occurring anxiety-like and aggressive behaviors induced by CCS.

Notably, CCKAR agonist increased, while antagonist decreased, the excitability of mPOA^Gad2^ neurons, consistently with previous findings that sulfated cholecystokinin octapeptide (CCK-8) depolarizes substantia nigra dopaminergic neurons in rats and increases AP firing frequency via CCKAR activation [65]. Furthermore, CCKAR may trigger intracellular calcium release through Gq-initiated signaling pathways [66] and CCK modulates the excitability of hippocampal interneurons by inhibiting Ca^2+^-activated K^+^ currents (IK_(Ca)_) [67]. Studying the specific signaling pathways or ionic mechanisms by which CCKAR modulates mPOA^Gad2^ neuronal excitability will expand our understanding of the delicate and complex interactions between neuropeptidergic and GABAergic systems.

In conclusion, our study showed that CCKAR in mPOA^Gad2^ neurons exert bidirectional control over anxiety-like and aggressive behaviors. The CCS paradigm introduced here provides a novel approach for exploring anxiety-like and aggressive behaviors induced by emotional stress. The mechanisms revealed in this study advance our understanding of the emotional regulatory role of CCKAR in the brain, identifying CCKAR in mPOA^Gad2^ neurons as a potential biomarker and therapeutic target for managing anxiety disorders associated with aggression.

## MATERIALS AND METHODS

### Mice

All procedures were approved by the Animal Care and Use Committee of the Animal Facility at Zhejiang University and were performed in accordance with the NIH Guideline for the Care and Use of Laboratory Animals. Adult male and female *C57BL/6J* mice (*C57* mice, purchased from Hangzhou Ziyuan Company), male Gad2-ires-Cre (JAX Strain#: 017320) and male Vglut2-ires-Cre (JAX Strain#: 028863) mice were used in this study. All transgenic mouse lines were bred on the *C57* background. Mice aged 8–12 weeks were used for the electrophysiological and behavioral experiments. All mice were socially housed, with three or four mice per cage. Mice were maintained at 23 ± 1°C and 40% humidity under a 12-h light/dark cycle (lights on from 7 AM to 7 PM every day) with food and water provided *ad libitum*. Behavioral and electrophysiological experiments were performed during the light phase.

Detailed materials and methods are available in the Supplementary data.

## Supplementary Material

nwaf152_Supplemental_Files

## Data Availability

All custom code used for analysis in this manuscript is available on request. All data are reported in the main text and supplementary materials, stored at Zhejiang University and available upon request.
