# Supplementary material for: Medial preoptic CCKAR mediates anxiety and aggression induced by chronic emotional stress in male mice
Source: Natl Sci Rev. 2025 May 21;12(10):nwaf152. doi: 10.1093/nsr/nwaf152 (PMC12418936; doi:10.1093/nsr/nwaf152)
Supplement: nwaf152_Supplemental_Files [file nwaf152_supplemental_files.zip › Supplemengary table_S1.pdf]

Figure	Description	Sample size	Statistic method	P value
Fig. 1B	CON vs CCS	n = 11, 11	Unpaired t test	*P* = 0.0083
Fig. 1C	CON vs CCS	n = 11, 11	Unpaired t test	*P* = 0.0108
Fig. 1D	CON vs CCS	n = 6, 8	Unpaired t test	*P* = 0.0006
Fig. 1F	CON vs CCS	n = 9, 9	Chi-square test with Fisher's exact test	*P* = 0.0004
Fig. 1G	CON vs CCS	n = 9, 9	Unpaired t test	*P* = 0.0001
Fig. 1H (left)	E vs S1 (CON)<br>E vs S1 (CCS)	n = 10<br>n = 11	Two-way ANOVA with Šídák's multiple comparisons	*P* < 0.0001<br>*P* < 0.0001
Fig. 1H (right)	CON vs CCS	n = 10, 11	Unpaired t test	*P* = 0.9862
Fig. 1I (left)	S1 vs S2 (CON)<br>S1 vs S2 (CCS)	n = 10<br>n = 11	Two-way ANOVA with Šídák's multiple comparisons	*P* = 0.0004<br>*P* = 0.2800
Fig. 1I (right)	CON vs CCS	n = 10, 11	Unpaired t test	*P* < 0.0001
Fig. 1J	CON vs CCS	n = 11, 11	Unpaired t test	*P* = 0.2573
Fig. 1K	CON vs CCS	n = 11, 11	Unpaired t test	*P* = 0.4060
Fig. 2B	CON vs CCS (mPOA)	n = 3 mice	Unpaired t test	*P* = 0.0062
	CON vs CCS (BNST)	n = 3 mice	Unpaired t test	*P* = 0.4127
	CON vs CCS (MeA)	n = 3 mice	Unpaired t test	*P* = 0.8491
	CON vs CCS (VMH)	n = 3 mice	Unpaired t test	*P* = 0.7841
Fig. 2D	CON vs CCS	n = 3 mice	Unpaired t test	*P* = 0.0318
Fig. 2F	CON vs CCS	n = 12 from 3 mice	Unpaired t test	*P* = 0.0186
Fig. 2G	CON vs CCS	n = 12 from 3 mice	Unpaired t test	*P* < 0.0001
Fig. 2I	$Gad2^{mcherry}$+Saline vs $Gad2^{mcherry}$+CNO<br>$Gad2^{hM4Di}$+Saline vs $Gad2^{hM4Di}$+CNO<br>$Gad2^{mcherry}$+Saline vs $Gad2^{hM4Di}$+Saline<br>$Gad2^{mcherry}$+CNO vs $Gad2^{hM4Di}$+CNO	n = 9, 12<br>n = 9, 12<br>n = 9, 9<br>n = 12, 12	Two-way ANOVA with Šídák's multiple comparisons	*P* = 0.9999<br>*P* = 0.0013<br>*P* = 0.9214<br>*P* < 0.0001
Fig. 2J	$Gad2^{mcherry}$+Saline vs $Gad2^{mcherry}$+CNO<br>$Gad2^{hM4Di}$+Saline vs $Gad2^{hM4Di}$+CNO<br>$Gad2^{mcherry}$+Saline vs $Gad2^{hM4Di}$+Saline<br>$Gad2^{mcherry}$+CNO vs $Gad2^{hM4Di}$+CNO	n = 9, 12<br>n = 9, 12<br>n = 9, 9<br>n = 12, 12	Two-way ANOVA with Šídák's multiple comparisons	*P* = 0.3003<br>*P* = 0.0017<br>*P* = 0.9985<br>*P* < 0.0001

Fig. 2L	Gad2^{mcherry}+Saline vs Gad2^{mcherry}+CNO<br>Gad2^{hM4Di}+Saline vs Gad2^{hM4Di}+CNO<br>Gad2^{mcherry}+Saline vs Gad2^{hM4Di}+Saline<br>Gad2^{mcherry}+CNO vs Gad2^{hM4Di}+CNO	n = 6, 6<br>n = 6, 6<br>n = 6, 6<br>n = 6, 6	Chi-square test with Fisher’s exact test	P = 0.0152<br>P = 0.0152<br>P = 0.0152<br>P = 0.0152
Fig. 2M	Gad2^{mcherry}+Saline vs Gad2^{mcherry}+CNO<br>Gad2^{hM4Di}+Saline vs Gad2^{hM4Di}+CNO<br>Gad2^{mcherry}+Saline vs Gad2^{hM4Di}+Saline<br>Gad2^{mcherry}+CNO vs Gad2^{hM4Di}+CNO	n = 6, 6<br>n = 6, 6<br>n = 6, 6<br>n = 6, 6	Two-way ANOVA with Šídák's multiple comparisons	P = 0.6493<br>P = 0.4961<br>P > 0.9999<br>P = 0.0280
Fig. 2N (left)	S1 vs S2 (Gad2^{mcherry}+Saline)<br>S1 vs S2 (Gad2^{mcherry}+CNO)<br>S1 vs S2 (Gad2^{hM4Di}+Saline)<br>S1 vs S2 (Gad2^{hM4Di}+CNO)	n = 9<br>n = 12<br>n = 9<br>n = 12	Two-way ANOVA with Šídák's multiple comparisons	P = 0.2194<br>P = 0.9874<br>P = 0.4030<br>P < 0.0001
Fig. 2N (right)	Gad2^{mcherry}+Saline vs Gad2^{mcherry}+CNO<br>Gad2^{hM4Di}+Saline vs Gad2^{hM4Di}+CNO<br>Gad2^{mcherry}+Saline vs Gad2^{hM4Di}+Saline<br>Gad2^{mcherry}+CNO vs Gad2^{hM5Di}+CNO	n = 9, 12<br>n = 9, 12<br>n = 9, 9<br>n = 12, 12	Two-way ANOVA with Šídák's multiple comparisons	P = 0.0732<br>P < 0.0001<br>P = 0.9981<br>P < 0.0001
Fig. 2P	Gad2^{mcherry}+Saline vs Gad2^{mcherry}+CNO<br>Gad2^{hM3Dq}+Saline vs Gad2^{hM3Dq}+CNO<br>Gad2^{mcherry}+Saline vs Gad2^{hM3Dq}+Saline<br>Gad2^{mcherry}+CNO vs Gad2^{hM3Dq}+CNO	n = 11, 11<br>n = 11, 12<br>n = 11, 11<br>n = 11, 12	Two-way ANOVA with Šídák's multiple comparisons	P = 0.0726<br>P < 0.0001<br>P = 0.8497<br>P < 0.0001
Fig. 2Q	Gad2^{mcherry}+Saline vs Gad2^{mcherry}+CNO<br>Gad2^{hM3Dq}+Saline vs Gad2^{hM3Dq}+CNO<br>Gad2^{mcherry}+Saline vs Gad2^{hM3Dq}+Saline<br>Gad2^{mcherry}+CNO vs Gad2^{hM3Dq}+CNO	n = 11, 11<br>n = 11, 12<br>n = 11, 11<br>n = 11, 12	Two-way ANOVA with Šídák's multiple comparisons	P = 0.6357<br>P = 0.0006<br>P = 0.0799<br>P = 0.0175
Fig. 2S	Gad2^{mcherry}+Saline vs Gad2^{mcherry}+CNO<br>Gad2^{hM3Dq}+Saline vs Gad2^{hM3Dq}+CNO<br>Gad2^{mcherry}+Saline vs Gad2^{hM3Dq}+Saline<br>Gad2^{mcherry}+CNO vs Gad2^{hM3Dq}+CNO	n = 6, 6<br>n = 6, 6<br>n = 6, 6<br>n = 6, 6	Chi-square test with Fisher’s exact test	P > 0.9999<br>P = 0.0801<br>P > 0.9999<br>P = 0.0152
Fig. 2T	Gad2^{mcherry}+Saline vs Gad2^{mcherry}+CNO<br>Gad2^{hM3Dq}+Saline vs Gad2^{hM3Dq}+CNO<br>Gad2^{mcherry}+Saline vs Gad2^{hM3Dq}+Saline<br>Gad2^{mcherry}+CNO vs Gad2^{hM3Dq}+CNO	n = 6, 6<br>n = 6, 6<br>n = 6, 6<br>n = 6, 6	Two-way ANOVA with Šídák's multiple comparisons	P > 0.9999<br>P = 0.0003<br>P > 0.9992<br>P = 0.0002
Fig. 2U (left)	S1 vs S2 (Gad2^{mcherry}+Saline)<br>S1 vs S2 (Gad2^{mcherry}+CNO)<br>S1 vs S2 (Gad2^{hM3Dq}+Saline)<br>S1 vs S2 (Gad2^{hM3Dq}+CNO)	n = 11<br>n = 11<br>n = 11<br>n = 12	Two-way ANOVA with Šídák's multiple comparisons	P = 0.0104<br>P = 0.0471<br>P = 0.0151<br>P < 0.5881

Fig. 2U (right)	$Gad2^{mcherry}$+Saline vs $Gad2^{mcherry}$+CNO<br>$Gad2^{hM3Dq}$+Saline vs $Gad2^{hM3Dq}$+CNO<br>$Gad2^{mcherry}$+Saline vs $Gad2^{hM3Dq}$+Saline<br>$Gad2^{mcherry}$+CNO vs $Gad2^{hM3Dq}$+CNO	n = 11, 11<br>n = 11, 12<br>n = 11, 11<br>n = 11, 12	Two-way ANOVA with Šídák's multiple comparisons	*P* = 0.5729<br>*P* = 0.0071<br>*P* = 0.7582<br>*P* = 0.0151
Fig. 3B (right)	CON vs CSS (CCKAR)<br>CON vs CSS (AR)<br>CON vs CSS (ESR1)<br>CON vs CSS (CALCR)	n = 6 each group	Two-way ANOVA with Šídák's multiple comparisons	*P* = 0.0163<br>*P* = 0.9779<br>*P* = 0.8408<br>*P* = 0.9931
Fig. 3D	CON vs CSS	n = 3 mice	Unpaired t test	*P* = 0.0400
Fig. 3F	*Gad2*$^{+}$/*Cckar*$^{+}$ vs *Slc17a6*$^{+}$/*Cckar*+	n = 3 mice	Unpaired t test	*P* < 0.0001
Fig. 3G	Persentage of *Gad2*$^{+}$*Cckar*$^{+}$ or	n = 3 mice		
Fig. 3I	EGFP vs sh*Cckar*	n = 4 mice	Unpaired t test	*P* < 0.0001
Fig. 3J	EGFP vs sh*Cckar*	n = 6, 5	Unpaired t test	*P* = 0.0012
Fig. 3K	EGFP vs sh*Cckar*	n = 11, 16	Unpaired t test	*P* = 0.0002
Fig. 3L	EGFP vs sh*Cckar*	n = 11, 16	Unpaired t test	*P* < 0.0001
Fig. 3N	EGFP vs sh*Cckar*	n = 6, 6	Chi-square test with Fisher's exact test	*P* = 0.0152
Fig. 3O	EGFP vs sh*Cckar*	n = 6, 6	Unpaired t test	*P* = 0.0091
Fig. 3P (left)	S1 vs S2 (EGFP)<br>S1 vs S2 (sh*Cckar*)	n = 11<br>n = 16	Two-way ANOVA with Šídák's multiple comparisons	*P* = 0.0285<br>*P* < 0.0001
Fig. 3P (right)	EGFP vs sh*Cckar*	n = 11, 16	Unpaired t test	*P* < 0.0001
Fig. 3Q	aCSF vs MK-329	n = 10, 8	Unpaired t test	*P* = 0.0002
Fig. 3R	aCSF vs MK-329	n = 10, 8	Unpaired t test	*P* = 0.0018
Fig. 3T	aCSF vs MK-329	n = 6, 6	Chi-square test with Fisher's exact test	*P* = 0.0152
Fig. 3U	aCSF vs MK-329	n = 6, 6	Unpaired t test	*P* = 0.0745
Fig. 3V (left)	S1 vs S2 (aCSF)<br>S1 vs S2 (MK-329)	n = 10<br>n = 8	Two-way ANOVA with Šídák's multiple comparisons	*P* = 0.0499<br>*P* = 0.0046
Fig. 3V (right)	aCSF vs MK-329	n = 10, 8	Unpaired t test	*P* < 0.0001
Fig. 4B	EGFP vs ov*Cckar*	n = 4, 4	Unpaired t test	*P* < 0.0001
Fig. 4C	EGFP vs ov*Cckar*	n = 3, 3	Unpaired t test	*P* = 0.0415
Fig. 4D	EGFP vs ov*Cckar*	n = 12, 12	Unpaired t test	*P* = 0.0021
Fig. 4E	EGFP vs ov*Cckar*	n = 12, 12	Unpaired t test	*P* = 0.0007
Fig. 4G	EGFP vs ov*Cckar*	n = 12, 12	Chi-square test with Fisher's exact test	*P* = 0.0152
Fig. 4H	EGFP vs ov*Cckar*	n = 12, 12	Unpaired t test	*P* = 0.0335

Fig. 4I (left)	S1 vs S2 (EGFP)<br>S1 vs S2 (ov*Cckar*)	n = 12<br>n = 12	Two-way ANOVA with Šídák's multiple comparisons	*P* < 0.0001<br>*P* = 0.0060
Fig. 4I (right)	EGFP vs ov*Cckar*	n = 12, 12	Unpaired t test	*P* < 0.0001
Fig. 4J	aCSF vs A71623	n = 12, 10	Unpaired t test	*P* = 0.0001
Fig. 4K	aCSF vs A71623	n = 12, 10	Unpaired t test	*P* = 0.0072
Fig. 4M	aCSF vs A71623	n = 6, 6	Chi-square test with Fisher’s exact test	*P* = 0.0152
Fig. 4N	aCSF vs A71623	n = 6, 6	Unpaired t test	*P* = 0.0048
Fig. 4O (left)	S1 vs S2 (aCSF)<br>S1 vs S2 (A71623)	n = 12<br>n = 10	Two-way ANOVA with Šídák's multiple comparisons	*P* = 0.0010<br>*P* < 0.0001
Fig. 4O (right)	aCSF vs A71623	n = 12, 10	Unpaired t test	*P* < 0.0001
Fig. 5C	CON vs CCS (pre)	n = 9 neurons	Unpaired t test	*P* = 0.0310
	pre vs post (CON+A71623)<br>pre vs wash (CON+A71623)<br>post vs wash (CON+A71623)	n = 9 neurons from 3 mice	Two-way ANOVA with Turkey's multiple comparisons	*P* = 0.0003<br>*P* = 0.2129<br>*P* = 0.0015
	pre vs post (CCS+MK-329)<br>pre vs wash (CCS+MK-329)<br>post vs wash (CCS+MK-329)	n = 9 neurons from 3 mice	Two-way ANOVA with Turkey's multiple comparisons	*P* = 0.0031<br>*P* = 0.0766<br>*P* = 0.0339
Fig. 5D	CON vs CCS (pre)	n = 9 neurons	Unpaired t test	*P* < 0.0001
	pre vs post (CON+A71623)<br>pre vs wash (CON+A71623)<br>post vs wash (CON+A71623)	n = 9 neurons from 3 mice	Two-way ANOVA with Turkey's multiple comparisons	*P* < 0.0001<br>*P* = 0.9607<br>*P* < 0.0001
	pre vs post (CCS+MK-329)<br>pre vs wash (CCS+MK-329)<br>post vs wash (CCS+MK-329)	n = 9 neurons from 3 mice	Two-way ANOVA with Turkey's multiple comparisons	*P* = 0.0022<br>*P* = 0.6466<br>*P* = 0.0006
Fig. 5E	CON vs CCS (pre)	n = 9 neurons	Unpaired t test	*P* = 0.1666
	pre vs post (CON+A71623)<br>pre vs wash (CON+A71623)<br>post vs wash (CON+A71623)	n = 9 neurons from 3 mice	Two-way ANOVA with Turkey's multiple comparisons	*P* = 0.9161<br>*P* = 0.3572<br>*P* = 0.1207
	pre vs post (CCS+MK-329)<br>pre vs wash (CCS+MK-329)<br>post vs wash (CCS+MK-329)	n = 9 neurons from 3 mice	Two-way ANOVA with Turkey's multiple comparisons	*P* = 0.4431<br>*P* > 0.9999<br>*P* = 0.0837
	CON vs CCS (pre)	n = 9 neurons	Unpaired t test	*P* = 0.6472

Fig. 5G	pre vs post (CON+A71623)<br>pre vs wash (CON+A71623)<br>post vs wash (CON+A71623)	n = 9 neurons from 3 mice	Two-way ANOVA with Turkey's multiple comparisons	*P* = 0.8852<br>*P* = 0.9172<br>*P* = 0.5343
	pre vs post (CCS+MK-329)<br>pre vs wash (CCS+MK-329)<br>post vs wash (CCS+MK-329)	n = 9 neurons from 3 mice	Two-way ANOVA with Turkey's multiple comparisons	*P* = 0.9347<br>*P* = 0.6764<br>*P* = 0.2828
Fig. 5H	CON vs CCS (pre)	n = 9 neurons	Unpaired t test	*P* = 0.0863
	pre vs post (CON+A71623)<br>pre vs wash (CON+A71623)<br>post vs wash (CON+A71623)	n = 9 neurons from 3 mice	Two-way ANOVA with Turkey's multiple comparisons	*P* = 0.1573<br>*P* = 0.9646<br>*P* = 0.0961
	pre vs post (CCS+MK-329)<br>pre vs wash (CCS+MK-329)<br>post vs wash (CCS+MK-329)	n = 9 neurons from 3 mice	Two-way ANOVA with Turkey's multiple comparisons	*P* = 0.3032<br>*P* = 0.8021<br>*P* = 0.0982
Fig. 5I	CON vs CCS (pre)	n = 9 neurons	Unpaired t test	*P* = 0.1818
	pre vs post (CON+A71623)<br>pre vs wash (CON+A71623)<br>post vs wash (CON+A71623)	n = 9 neurons from 3 mice	Two-way ANOVA with Turkey's multiple comparisons	*P* = 0.5006<br>*P* = 0.4871<br>*P* = 0.0505
	pre vs post (CCS+MK-329)<br>pre vs wash (CCS+MK-329)<br>post vs wash (CCS+MK-329)	n = 9 neurons from 3 mice	Two-way ANOVA with Turkey's multiple comparisons	*P* = 0.9081<br>*P* = 0.8422<br>*P* = 0.8121
Fig. S1B	no single vs female single<br>no single vs male single<br>female single vs male single	n = 10, 10<br>n = 10, 10<br>n = 10, 10	One-way ANOVA with Turkey's multiple comparisons	*P* = 0.2327<br>*P* = 0.9262<br>*P* = 0.4062
Fig. S1C	no single vs female single<br>no single vs male single<br>female single vs male single	n = 10, 10<br>n = 10, 10<br>n = 10, 10	One-way ANOVA with Turkey's multiple comparisons	*P* = 0.7437<br>*P* = 0.9709<br>*P* = 0.8693
Fig. S1D (left)	E vs S1 (no single)<br>E vs S1 (female single)<br>E vs S1 (male single)	n = 10<br>n = 10<br>n = 10	Two-way ANOVA with Šídák's multiple comparisons	*P* < 0.0001<br>*P* < 0.0001<br>*P* < 0.0001
Fig. S1D (right)	no single vs female single<br>no single vs male single<br>female single vs male single	n = 10, 10<br>n = 10, 10<br>n = 10, 10	One-way ANOVA with Turkey's multiple comparisons	*P* = 0.3617<br>*P* = 0.0717<br>*P* = 0.6318
Fig. S1E (left)	S1 vs S2 (no single)<br>S1 vs S2 (female single)<br>S1 vs S2 (male single)	n = 10<br>n = 10<br>n = 10	Two-way ANOVA with Šídák's multiple comparisons	*P* = 0.0011<br>*P* = 0.0006<br>*P* = 0.0094

Fig. S1E (right)	no single vs female single<br>no single vs male single<br>female single vs male single	n = 10, 10<br>n = 10, 10<br>n = 10, 10	One-way ANOVA with Turkey's multiple comparisons	*P* = 0.9054<br>*P* = 0.9679<br>*P* = 0.7833
Fig. S2B	CON vs (male partner, male single)<br>CON vs (female partner, female single)<br>(male partner, male single) vs (female partner, female single)	n = 9, 10<br>n = 10, 10<br>n = 10, 10	One-way ANOVA with Turkey's multiple comparisons	*P* = 0.3970<br>*P* = 0.9962<br>*P* = 0.4235
Fig. S2C	CON vs (male partner, male single)<br>CON vs (female partner, female single)<br>(male partner, male single) vs (female partner, female single)	n = 9, 10<br>n = 9, 10<br>n = 10, 10	One-way ANOVA with Turkey's multiple comparisons	*P* = 0.9577<br>*P* = 0.7899<br>*P* = 0.9208
Fig. S2D (left)	E vs S1 (CON)<br>E vs S1 (male partner, male single)<br>E vs S1 (female partner, female single)	n = 9<br>n = 10<br>n = 10	Two-way ANOVA with Šídák's multiple comparisons	*P* < 0.0001<br>*P* < 0.0001<br>*P* < 0.0001
Fig. S2D (right)	CON vs (male partner, male single)<br>CON vs (female partner, female single)<br>(male partner, male single) vs (female partner, female single)	n = 9, 10<br>n = 9, 10<br>n = 10, 10	One-way ANOVA with Dunnett's multiple comparisons	*P* = 0.9729<br>*P* = 0.0589<br>*P* = 0.0825
Fig. S2E (left)	S1 vs S2 (CON)<br>S1 vs S2 (male-partner paired)<br>S1 vs S2 (female single)	n = 9<br>n = 10<br>n = 10	Two-way ANOVA with Šídák's multiple comparisons	*P* = 0.0002<br>*P* < 0.0001<br>*P* = 0.0019
Fig. S2E (right)	CON vs (male partner, male single)<br>CON vs (female partner, female single)<br>(male partner, male single) vs (female partner, female single)	n = 9, 10<br>n = 9, 10<br>n = 10, 10	One-way ANOVA with Turkey's multiple comparisons	*P* = 0.6093<br>*P* = 0.2816<br>*P* = 0.8127
Fig. S2G	CON vs toy single<br>CON vs juvenile single	n = 10, 11<br>n = 10, 11	One-way ANOVA with Dunnett's multiple comparisons	*P* = 0.7659<br>*P* = 0.0630
Fig. S2H	CON vs toy single<br>CON vs juvenile single	n = 10, 11<br>n = 10, 11	One-way ANOVA with Dunnett's multiple comparisons	*P* = 0.6993<br>*P* > 0.9999
Fig. S2I (left)	E vs S1 (CON)<br>E vs S1 (toy single)<br>E vs S1 (juvenile single)	n = 10<br>n = 11<br>n = 11	Two-way ANOVA with Šídák's multiple comparisons	*P* < 0.0001<br>*P* < 0.0001<br>*P* < 0.0001
Fig. S2I (right)	CON vs toy single<br>CON vs juvenile single	n = 10, 11<br>n = 10, 11	One-way ANOVA with Dunnett's multiple comparisons	*P* = 0.3106<br>*P* = 0.5083

Fig. S2J (left)	S1 vs S2 (CON)<br>S1 vs S2 (toy single)<br>S1 vs S2 (juvenile single)	n = 10<br>n = 11<br>n = 11	Two-way ANOVA with Šídák's multiple comparisons	*P* < 0.0001<br>*P* < 0.0001<br>*P* < 0.0001
Fig. S2J (right)	CON vs toy single<br>CON vs juvenile single	n = 10, 11<br>n = 10, 11	One-way ANOVA with Dunnett's multiple comparisons	*P* = 0.1171<br>*P* = 0.0754
Fig. S3A	S-CON vs S-CCS<br>Z-CON vs Z-CCS<br>S-CON vs Z-CON<br>S-CCS vs Z-CCS	n = 11, 11<br>n = 10, 12<br>n = 11, 10<br>n = 11, 12	Two-way ANOVA with Šídák's multiple comparisons	*P* = 0.0097<br>*P* > 0.9999<br>*P* = 0.9845<br>*P* = 0.0312
Fig. S3B	S-CON vs S-CCS<br>Z-CON vs Z-CCS<br>S-CON vs Z-CON<br>S-CCS vs Z-CCS	n = 11, 11<br>n = 10, 12<br>n = 11, 10<br>n = 11, 12	Two-way ANOVA with Šídák's multiple comparisons	*P* = 0.0010<br>*P* > 0.9999<br>*P* = 0.6383<br>*P* < 0.0001
Fig. S3D	S-CON vs S-CCS<br>Z-CON vs Z-CCS<br>S-CON vs Z-CON<br>S-CCS vs Z-CCS	n = 6, 6<br>n = 6, 6<br>n = 6, 6<br>n = 6, 6	Chi-square test with Fisher's exact test	P = 0.0801<br>P > 0.9999<br>P > 0.9999<br>P = 0.0152
Fig. S3E	S-CON vs S-CCS<br>Z-CON vs Z-CCS<br>S-CON vs Z-CON<br>S-CCS vs Z-CCS	n = 6, 6<br>n = 6, 6<br>n = 6, 6<br>n = 6, 6	Two-way ANOVA with Šídák's multiple comparisons	*P* = 0.9649<br>*P* = 0.0053<br>*P* = 0.0341<br>*P* > 0.9999
Fig. S3F (left)	E vs S1 (S-CON)<br>E vs S1 (S-CCS)<br>E vs S1 (Z-CON)<br>E vs S1 (Z-CCS)	n = 11<br>n = 11<br>n = 10<br>n = 12	Two-way ANOVA with Šídák's multiple comparisons	*P* < 0.0001<br>*P* < 0.0001<br>*P* = 0.0013<br>*P* < 0.0001
Fig. S3F (right)	S-CON vs S-CCS<br>Z-CON vs Z-CCS<br>S-CON vs Z-CON<br>S-CCS vs Z-CCS	n = 11, 11<br>n = 11, 11<br>n = 10, 10<br>n = 12, 12	Two-way ANOVA with Šídák's multiple comparisons	*P* = 0.9862<br>*P* = 0.3318<br>*P* = 0.0515<br>*P* > 0.9999
Fig. S3G (left)	S1 vs S2 (S-CON)<br>S1 vs S2 (S-CCS)<br>S1 vs S2 (Z-CON)<br>S1 vs S2 (Z-CCS)	n = 11<br>n = 11<br>n = 10<br>n = 12	Two-way ANOVA with Šídák's multiple comparisons	*P* = 0.0026<br>*P* = 0.4983<br>*P* = 0.0024<br>*P* = 0.0053
Fig. S3G (right)	S-CON vs S-CCS<br>Z-CON vs Z-CCS<br>S-CON vs Z-CON<br>S-CCS vs Z-CCS	n = 11, 11<br>n = 11, 11<br>n = 10, 10<br>n = 12, 12	Two-way ANOVA with Šídák's multiple comparisons	*P* < 0.0001<br>*P* = 0.3056<br>*P* = 0.0374<br>*P* < 0.0001
Fig. S3H	S-CON vs S-CCS<br>O-CON vs O-CCS<br>S-CON vs O-CON<br>S-CCS vs O-CCS	n = 10, 10<br>n = 7, 9<br>n = 10, 7<br>n = 10, 9	Two-way ANOVA with Šídák's multiple comparisons	*P* = 0.0140<br>*P* = 0.5019<br>*P* = 0.6554<br>*P* > 0.9999
Fig. S3I	S-CON vs S-CCS<br>O-CON vs O-CCS<br>S-CON vs O-CON<br>S-CCS vs O-CCS	n = 10, 10<br>n = 7, 9<br>n = 10, 7<br>n = 10, 9	Two-way ANOVA with Šídák's multiple comparisons	*P* = 0.0059<br>*P* = 0.6287<br>*P* = 0.5219<br>*P* > 0.9999

Fig. S3K	S-CON vs S-CCS<br>O-CON vs O-CCS<br>S-CON vs O-CON<br>S-CCS vs O-CCS	n = 6, 6<br>n = 6, 6<br>n = 6, 6<br>n = 6, 6	Chi-square test with Fisher’s exact test	P = 0.0152<br>P =0.1833<br>P > 0.9999<br>P = 0.5455
Fig. S3L	S-CON vs S-CCS<br>O-CON vs O-CCS<br>S-CON vs O-CON<br>S-CCS vs O-CCS	n = 6, 6<br>n = 6, 6<br>n = 6, 6<br>n = 6, 6	Two-way ANOVA with Šídák's multiple comparisons	*P* = 0.0308<br>*P* = 0.1776<br>*P* > 0.9999<br>*P* = 0.9600
Fig. S3M (left)	E vs S1 (S-CON)<br>E vs S1 (S-CCS)<br>E vs S1 (O-CON)<br>E vs S1 (O-CCS)	n = 10<br>n = 10<br>n = 7<br>n = 9	Two-way ANOVA with Šídák's multiple comparisons	*P* < 0.0001<br>*P* < 0.0001<br>*P* < 0.0001<br>*P* < 0.0001
Fig. S3M (right)	S-CON vs S-CCS<br>O-CON vs O-CCS<br>S-CON vs O-CON<br>S-CCS vs O-CCS	n = 10, 10<br>n = 7, 9<br>n = 10, 7<br>n = 10, 9	Two-way ANOVA with Šídák's multiple comparisons	*P* = 0.4117<br>*P* > 0.9999<br>*P* = 0.8576<br>*P* = 0.9965
Fig. S3N (left)	S1 vs S2 (S-CON)<br>S1 vs S2 (S-CCS)<br>S1 vs S2 (O-CON)<br>S1 vs S2 (O-CCS)	n = 10<br>n = 10<br>n = 7<br>n = 9	Two-way ANOVA with Šídák's multiple comparisons	*P* < 0.0001<br>*P* = 0.0001<br>*P* = 0.0010<br>*P* = 0.0004
Fig. S3N (right)	S-CON vs S-CCS<br>O-CON vs O-CCS<br>S-CON vs O-CON<br>S-CCS vs O-CCS	n = 10, 10<br>n = 7, 9<br>n = 10, 7<br>n = 10, 9	Two-way ANOVA with Šídák's multiple comparisons	*P* < 0.0001<br>*P* < 0.0001<br>*P* = 0.0543<br>*P* = 0.9029
Fig. S3O	CON vs CCS	n = 11, 11	Unpaired t test	*P* = 0.0004
Fig. S3P	CON vs CCS	n = 11, 11	Unpaired t test	*P* < 0.0001
Fig. S3R	G-CON vs G-CCS	n = 6, 6	Chi-square test with Fisher’s exact test	*P* = 0.0152
Fig. S3S	G-CON vs G-CCS	n = 6, 6	Unpaired t test	*P* = 0.0089
Fig. S3T (left)	E vs S1 (G-CON)<br>E vs S1 (G-CCS)	n = 11<br>n = 11	Two-way ANOVA with Šídák's multiple comparisons	*P* < 0.0001<br>*P* < 0.0001
Fig. S3T (right)	G-CON vs G-CCS	n = 11, 11	Unpaired t test	*P* = 0.7635
Fig. S3U (left)	S1 vs S2 (G-CON)<br>S1 vs S2 (G-CCS)	n = 11<br>n = 11	Two-way ANOVA with Šídák's multiple comparisons	*P* = 0.0002<br>*P* = 0.0014
Fig. S3U (right)	G-CON vs G-CCS	n = 11, 11	Unpaired t test	*P* < 0.0001
Fig. S4B	CON vs CCS	n = 3, 3	Unpaired t test	*P* = 0.1459
Fig. S4E	CON vs CCS	n = 12 neurons from 3 mice	Unpaired t test	*P* = 0.9254
Fig. S4F	CON vs CCS		Unpaired t test	*P* = 0.3284
Fig. S4G	CON vs CCS		Unpaired t test	*P* = 0.1694
Fig. S4H	CON vs CCS		Unpaired t test	*P* = 0.8212
Fig. S4I	aCSF vs CNO	n=7 neurons from 2 mice	Unpaired t test	*P* < 0.0001

Fig. S4J	aCSF vs CNO	n=6 neurons from 2 mice	Unpaired t test	P = 0.0102
Fig. S4L	Saline vs DCZ	n = 10, 10	Unpaired t test	P = 0.0060
Fig. S4M	Saline vs DCZ	n = 10, 10	Unpaired t test	P < 0.0001
Fig. S4O	Saline vs DCZ	n = 6, 6	Chi-square test with Fisher's exact test	P = 0.0152
Fig. S4P	Saline vs DCZ	n = 6, 6	Unpaired t test	P = 0.0073
Fig. S4Q (left)	S1 vs S2 (Saline)<br>S1 vs S2 (DCZ)	n = 10<br>n = 10	Two-way ANOVA with Šídák's multiple comparisons	P < 0.0001<br>P < 0.0001
Fig. S4Q (right)	Saline vs DCZ	n = 10, 10	Unpaired t test	P < 0.0001
Fig. S4S	mcherry vs hM4Di	n = 8, 6	Unpaired t test	P = 0.4914
Fig. S4T	mcherry vs hM4Di	n = 8, 6	Unpaired t test	P = 0.7300
Fig. S4U (left)	E vs S1 (mcherry)<br>E vs S1 (hM4Di)	n = 8<br>n = 6	Two-way ANOVA with Šídák's multiple comparisons	P < 0.0001<br>P < 0.0001
Fig. S4U (right)	mcherry vs hM4Di	n = 8, 6	Unpaired t test	P = 0.1299
Fig. S4V (left)	S1 vs S2 (mcherry)<br>S1 vs S2 (hM4Di)	n = 8<br>n = 6	Two-way ANOVA with Šídák's multiple comparisons	P = 0.9909<br>P = 0.1705
Fig. S4V (right)	mcherry vs hM4Di	n = 8, 6	Unpaired t test	P = 0.0349
Fig. S5B	CON vs CCS	n = 12 from 3 mice	Unpaired t test	P = 0.0689
Fig. S5C	CON vs CCS		Unpaired t test	P = 0.6864
Fig. S5D	CON vs CCS		Unpaired t test	P = 0.5809
Fig. S5E	CON vs CCS		Unpaired t test	P = 0.1514
Fig. S5F	CON vs CCS		Unpaired t test	P = 0.9055
Fig. S5G	CON vs CCS		Unpaired t test	P = 0.2266
Fig. S5I	vGluT2^{mcherry}+Saline vs vGluT2^{mcherry}+CNO<br>vGluT2^{hM4Di}+Saline vs vGluT2^{hM4Di}+CNO<br>vGluT2^{mcherry}+Saline vs vGluT2^{hM4Di}+Saline<br>vGluT2^{mcherry}+CNO vs vGluT2$^{hM4Di+CNO}$	n = 9, 9<br>n = 9, 11<br>n = 9, 9<br>n = 9, 11	Two-way ANOVA with Šídák's multiple comparisons	P = 0.9832<br>P = 0.0771<br>P > 0.9999<br>P = 0.4275
Fig. S5J	vGluT2^{mcherry}+Saline vs vGluT2^{mcherry}+CNO<br>vGluT2^{hM4Di}+Saline vs vGluT2^{hM4Di}+CNO<br>vGluT2^{mcherry}+Saline vs vGluT2^{hM4Di}+Saline<br>vGluT2^{mcherry}+CNO vs vGluT2^{hM4Di}+CNO	n = 9, 9<br>n = 9, 11<br>n = 9, 9<br>n = 9, 11	Two-way ANOVA with Šídák's multiple comparisons	P = 0.8795<br>P = 0.3133<br>P = 0.9983<br>P = 0.7407
Fig. S5L	vGluT2^{mcherry}+Saline vs vGluT2^{mcherry}+CNO<br>vGluT2^{hM4Di}+Saline vs vGluT2^{hM4Di}+CNO<br>vGluT2^{mcherry}+Saline vs vGluT2^{hM4Di}+Saline<br>vGluT2^{mcherry}+CNO vs vGluT2^{hM4Di}+CNO	n = 6, 6<br>n = 6, 6<br>n = 6, 6<br>n = 6, 6	Chi-square test with Fisher's exact test	P > 0.9999<br>P > 0.9999<br>P > 0.9999<br>P > 0.9999

Fig. S5M	vGluT2^{mcherry}+Saline vs vGluT2^{mcherry}+CNO<br>vGluT2^{hM4Di}+Saline vs vGluT2^{hM4Di}+CNO<br>vGluT2^{mcherry}+Saline vs vGluT2^{hM4Di}+Saline<br>vGluT2^{mcherry}+CNO vs vGluT2^{hM4Di}+CNO	n = 6, 6<br>n = 6, 6<br>n = 6, 6<br>n = 6, 6	Two-way ANOVA with Šídák's multiple comparisons	*P* = 0.9995<br>*P* > 0.9999<br>*P* = 0.9960<br>*P* = 0.9895
Fig. S5N (left)	S1 vs S2 (vGluT2^{mcherry}+Saline)<br>S1 vs S2 (vGluT2^{mcherry}+CNO)<br>S1 vs S2 (vGluT2^{hM4Di}+Saline)<br>S1 vs S2 (vGluT2^{hM4Di}+CNO)	n = 9<br>n = 9<br>n = 9<br>n = 11	Two-way ANOVA with Šídák's multiple comparisons	*P* = 0.0013<br>*P* = 0.0027<br>*P* = 0.0409<br>*P* < 0.0001
Fig. S5N (right)	vGluT2^{mcherry}+Saline vs vGluT2^{mcherry}+CNO<br>vGluT2^{hM4Di}+Saline vs vGluT2^{hM4Di}+CNO<br>vGluT2^{mcherry}+Saline vs vGluT2^{hM4Di}+Saline<br>vGluT2^{mcherry}+CNO vs vGluT2$^{hM4Di+CNO}$	n = 9, 9<br>n = 9, 11<br>n = 9, 9<br>n = 9, 11	Two-way ANOVA with Šídák's multiple comparisons	*P* = 0.5927<br>*P* > 0.9999<br>*P* = 0.9958<br>*P* = 0.8946
Fig. S6A	CON vs SCS	n = 11, 10	Unpaired t test	*P* = 0.1950
Fig. S6B	CON vs SCS	n = 11, 10	Unpaired t test	*P* = 0.5440
Fig. S6D	CON vs SCS	n = 6, 6	Chi-square test with Fisher's exact test	*P* = 0.2424
Fig. S6E	CON vs SCS	n = 6, 6	Unpaired t test	*P* = 0.0804
Fig. S6F (left)	E vs S1 (CON)<br>E vs S1 (SCS)	n = 11<br>n = 10	Two-way ANOVA with Šídák's multiple comparisons	*P* < 0.0001<br>*P* < 0.0001
Fig. S6F (right)	CON vs SCS	n = 11, 10	Unpaired t test	*P* = 0.3704
Fig. S6G (left)	S1 vs S2 (CON)<br>S1 vs S2 (SCS)	n = 11<br>n = 10	Two-way ANOVA with Šídák's multiple comparisons	*P* < 0.0001<br>*P* < 0.0001
Fig. S6G (right)	CON vs SCS	n = 11, 10	Unpaired t test	*P* = 0.7079
Fig. S7B	CON vs CCS (BNST)	n = 3 mice	Unpaired t test	*P* = 0.3165
	CON vs CCS (MeA)	n = 3 mice	Unpaired t test	*P* = 0.2638
	CON vs CCS (VMH)	n = 3 mice	Unpaired t test	*P* = 0.8699
Fig. S7C	Male vs Female	n = 3 mice	Unpaired t test	*P* = 0.0009
Fig. S7E	Gad2^{+}/*Cckar*$^{+}$ vs Slc17a6^{+}/*Cckar*+	n = 3 mice	Unpaired t test	*P* = 0.0052
Fig. S8B	EGFP vs sh*Cckar*	n = 11, 10	Unpaired t test	*P* = 0.4441
Fig. S8C	EGFP vs sh*Cckar*	n = 11, 10	Unpaired t test	*P* = 0.9717
Fig. S8E	EGFP vs sh*Cckar*	n = 6, 6	Chi-square test with Fisher's exact test	*P* > 0.9999
Fig. S8F	EGFP vs sh*Cckar*	n = 6, 6	Unpaired t test	*P* = 0.6871

Fig. S8G (left)	E vs S1 (EGFP)<br>E vs S1 (sh*Cckar*)	n = 11<br>n = 10	Two-way ANOVA with Šídák's multiple comparisons	*P* < 0.0001<br>*P* < 0.0001
Fig. S8G (right)	EGFP vs sh*Cckar*	n = 11, 10	Unpaired t test	*P* = 0.5856
Fig. S8H (left)	S1 vs S2 (EGFP)<br>S1 vs S2 (sh*Cckar*)	n = 11<br>n = 10	Two-way ANOVA with Šídák's multiple comparisons	*P* = 0.9625<br>*P* = 0.9509
Fig. S8H (right)	EGFP vs sh*Cckar*	n = 11, 10	Unpaired t test	*P* = 0.9534
Fig. S8J	EGFP vs sh*Cckar*	n = 11, 10	Unpaired t test	*P* = 0.7226
Fig. S8K	EGFP vs sh*Cckar*	n = 11, 10	Unpaired t test	*P* = 0.0122
Fig. S8M	EGFP vs sh*Cckar*	n = 6, 6	Chi-square test with Fisher's exact test	*P* > 0.9999
Fig. S8N	EGFP vs sh*Cckar*	n = 6, 6	Unpaired t test	*P* = 0.3426
Fig. S8O (left)	E vs S1 (EGFP)<br>E vs S1 (sh*Cckar*)	n = 11<br>n = 10	Two-way ANOVA with Šídák's multiple comparisons	*P* < 0.0001<br>*P* < 0.0001
Fig. S8O (right)	EGFP vs sh*Cckar*	n = 11, 10	Unpaired t test	*P* = 0.9534
Fig. S8P (left)	S1 vs S2 (EGFP)<br>S1 vs S2 (shCckar)	n = 11<br>n = 10	Two-way ANOVA with Šídák's multiple comparisons	*P* = 0.9625<br>*P* = 0.9509
Fig. S8P (right)	EGFP vs sh*Cckar*	n = 11, 10	Unpaired t test	P = 0.6892
Fig. S9B	CON-EGFP vs SSDS-EGFP<br>CON-EGFP vs SSDS-shCckar<br>SSDS-EGFP vs SSDS-shCckar	n = 8, 9<br>n = 8, 12<br>n = 9, 12	One-way ANOVA with Turkey's multiple comparisons	*P* = 0.0007<br>*P* = 0.0082<br>*P* = 0.4241
Fig. S9C	CON-EGFP vs SSDS-EGFP<br>CON-EGFP vs SSDS-shCckar<br>SSDS-EGFP vs SSDS-shCckar	n = 8, 9<br>n = 8, 12<br>n = 9, 12	One-way ANOVA with Turkey's multiple comparisons	*P* = 0.0308<br>*P* = 0.0027<br>*P* = 0.6644
Fig. S9D (left)	E vs S1 (CON-EGFP)<br>E vs S1 (SSDS-EGFP)<br>E vs S1 (SSDS-shCckar)	n = 8<br>n = 9<br>n = 12	Two-way ANOVA with Šídák's multiple comparisons	*P* < 0.0001<br>*P* < 0.0001<br>*P* < 0.0001
Fig. S9D (right)	CON-EGFP vs SSDS-EGFP<br>CON-EGFP vs SSDS-shCckar<br>SSDS-EGFP vs SSDS-shCckar	n = 8, 9<br>n = 8, 12<br>n = 9, 12	One-way ANOVA with Turkey's multiple comparisons	*P* = 0.2245<br>*P* = 0.1085<br>*P* = 0.9455
Fig. S9E (left)	S1 vs S2 (CON-EGFP)<br>S1 vs S2 (SSDS-EGFP)<br>S1 vs S2 (SSDS-shCckar)	n = 11<br>n = 10	Two-way ANOVA with Šídák's multiple comparisons	*P* = 0.0071<br>*P* = 0.0006<br>*P* = 0.2870
Fig. S9E (right)	CON-EGFP vs SSDS-EGFP<br>CON-EGFP vs SSDS-shCckar<br>SSDS-EGFP vs SSDS-shCckar	n = 8, 9<br>n = 8, 12<br>n = 9, 12	One-way ANOVA with Turkey's multiple comparisons	*P* = 0.6697<br>*P* = 0.7522<br>*P* = 0.2257

Fig. S9F SPT	CON-EGFP vs SSDS-EGFP<br>CON-EGFP vs SSDS-shCckar<br>SSDS-EGFP vs SSDS-shCckar	n = 8, 9<br>n = 8, 12<br>n = 9, 12	One-way ANOVA with Turkey's multiple comparisons	*P* = 0.9942<br>*P* = 0.4341<br>*P* = 0.3506
Fig. S9G FST	CON-EGFP vs SSDS-EGFP<br>CON-EGFP vs SSDS-shCckar<br>SSDS-EGFP vs SSDS-shCckar	n = 8, 9<br>n = 8, 12<br>n = 9, 12	One-way ANOVA with Turkey's multiple comparisons	*P* = 0.9971<br>*P* = 0.4514<br>*P* = 0.4724
Fig. S10A	CON vs CCS (pre)	n = 9 neurons	Unpaired t test	*P* = 0.2059
	pre vs post (CON+A71623)<br>pre vs wash (CON+A71623)<br>post vs wash (CON+A71623)	n = 9 neurons from 3 mice	Two-way ANOVA with Turkey's multiple comparisons	*P* = 0.1319<br>*P* = 0.0758<br>*P* = 0.7614
	pre vs post (CCS+MK-329)<br>pre vs wash (CCS+MK-329)<br>post vs wash (CCS+MK-329)	n = 9 neurons from 3 mice	Two-way ANOVA with Turkey's multiple comparisons	*P* = 0.2728<br>*P* = 0.3351<br>*P* = 0.9889
Fig. S10B	CON vs CCS (pre)	n = 9 neurons	Unpaired t test	*P* = 0.7767
	pre vs post (CON+A71623)<br>pre vs wash (CON+A71623)<br>post vs wash (CON+A71623)	n = 9 neurons from 3 mice	Two-way ANOVA with Turkey's multiple comparisons	*P* = 0.2758<br>*P* = 0.2477<br>*P* = 0.8082
	pre vs post (CCS+MK-329)<br>pre vs wash (CCS+MK-329)<br>post vs wash (CCS+MK-329)	n = 9 neurons from 3 mice	Two-way ANOVA with Turkey's multiple comparisons	*P* > 0.9999<br>*P* = 0.2454<br>*P* = 0.2892
Fig. S10C	CON vs CCS (pre)	n = 9 neurons	Unpaired t test	*P* = 0.2126
	pre vs post (CON+A71623)<br>pre vs wash (CON+A71623)<br>post vs wash (CON+A71623)	n = 9 neurons from 3 mice	Two-way ANOVA with Turkey's multiple comparisons	*P* = 0.5487<br>*P* = 0.3210<br>*P* = 0.8220
	pre vs post (CCS+MK-329)<br>pre vs wash (CCS+MK-329)<br>post vs wash (CCS+MK-329)	n = 9 neurons from 3 mice	Two-way ANOVA with Turkey's multiple comparisons	*P* = 0.9123<br>*P* = 0.1878<br>*P* = 0.1126
Fig. S10D	CON vs CCS (pre)	n = 9 neurons	Unpaired t test	*P* = 0.2718
	pre vs post (CON+A71623)<br>pre vs wash (CON+A71623)<br>post vs wash (CON+A71623)	n = 9 neurons from 3 mice	Two-way ANOVA with Turkey's multiple comparisons	*P* = 0.2189<br>*P* = 0.3452<br>*P* = 0.7980
	pre vs post (CCS+MK-329)<br>pre vs wash (CCS+MK-329)<br>post vs wash (CCS+MK-329)	n = 9 neurons from 3 mice	Two-way ANOVA with Turkey's multiple comparisons	*P* = 0.2027<br>*P* = 0.1148<br>*P* = 0.4268
	CON vs CCS (pre)	n = 9 neurons	Unpaired t test	*P* = 0.3284

Fig. S10E	pre vs post (CON+A71623)<br>pre vs wash (CON+A71623)<br>post vs wash (CON+A71623)	n = 9 neurons from 3 mice	Two-way ANOVA with Turkey's multiple comparisons	*P* = 0.0613<br>*P* = 0.4557<br>*P* = 0.9777
	pre vs post (CCS+MK-329)<br>pre vs wash (CCS+MK-329)<br>post vs wash (CCS+MK-329)	n = 9 neurons from 3 mice	Two-way ANOVA with Turkey's multiple comparisons	*P* = 0.1684<br>*P* = 0.4657<br>*P* = 0.9847
Fig. S10F	CON vs CCS (pre)	n = 9 neurons	Unpaired t test	*P* = 0.5465
	pre vs post (CON+A71623)<br>pre vs wash (CON+A71623)<br>post vs wash (CON+A71623)	n = 9 neurons from 3 mice	Two-way ANOVA with Turkey's multiple comparisons	P = 0.0613<br>P = 0.4557<br>P = 0.9777
	pre vs post (CCS+MK-329)<br>pre vs wash (CCS+MK-329)<br>post vs wash (CCS+MK-329)	n = 9 neurons from 3 mice	Two-way ANOVA with Turkey's multiple comparisons	P = 0.1684<br>P = 0.4657<br>P = 0.9847
Fig. S11B	Persentage of *Gad2*$^{+}$*slc17a6*$^{+}$ or *Gad2*$^{+}$*slc17a6*$^{-}$	n = 3 mice		
Fig. S11D	*Cckar*$^{+}$*Gad2*$^{+}$/*Gad2*$^{+}$ or	n = 3 mice		
Fig. S11E	*Esr1*$^{+}$*Cckar*$^{+}$/*Cckar*$^{+}$ or	n = 3 mice		
