# Supplementary material for: Medial preoptic CCKAR mediates anxiety and aggression induced by chronic emotional stress in male mice
Source: Natl Sci Rev. 2025 May 21;12(10):nwaf152. doi: 10.1093/nsr/nwaf152 (PMC12418936; doi:10.1093/nsr/nwaf152)
Supplement: nwaf152_Supplemental_Files [file nwaf152_supplemental_files.zip › Supplementary figure files.docx]

**SUPPLEMENTARY FIGURES**

**
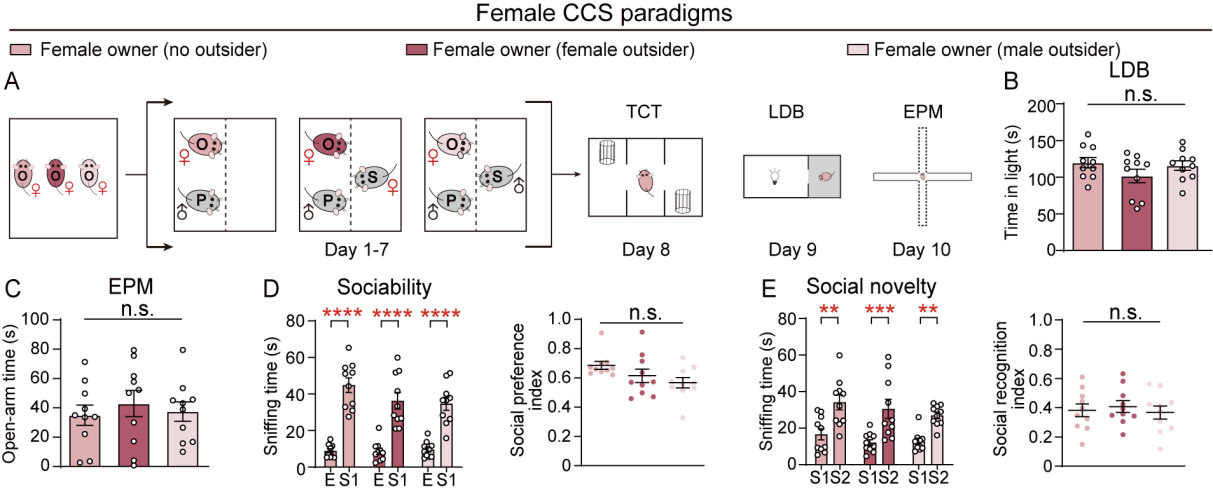
**

**Fig. S1. Female mice do not respond to CCS paradigm.**

(A) Paradigm for modified female CCS model. (B, E) Quantification of time spent in light chamber in the LDB (B), time spent in open arms in the EPM (C), and sniffing time and social indexes in TCT (D, E) Values are means ± SEM. ***P* < 0.01, ****P* < 0.001, *****P* < 0.0001, n.s., no significance (see table S1 for statistics and n numbers).

**
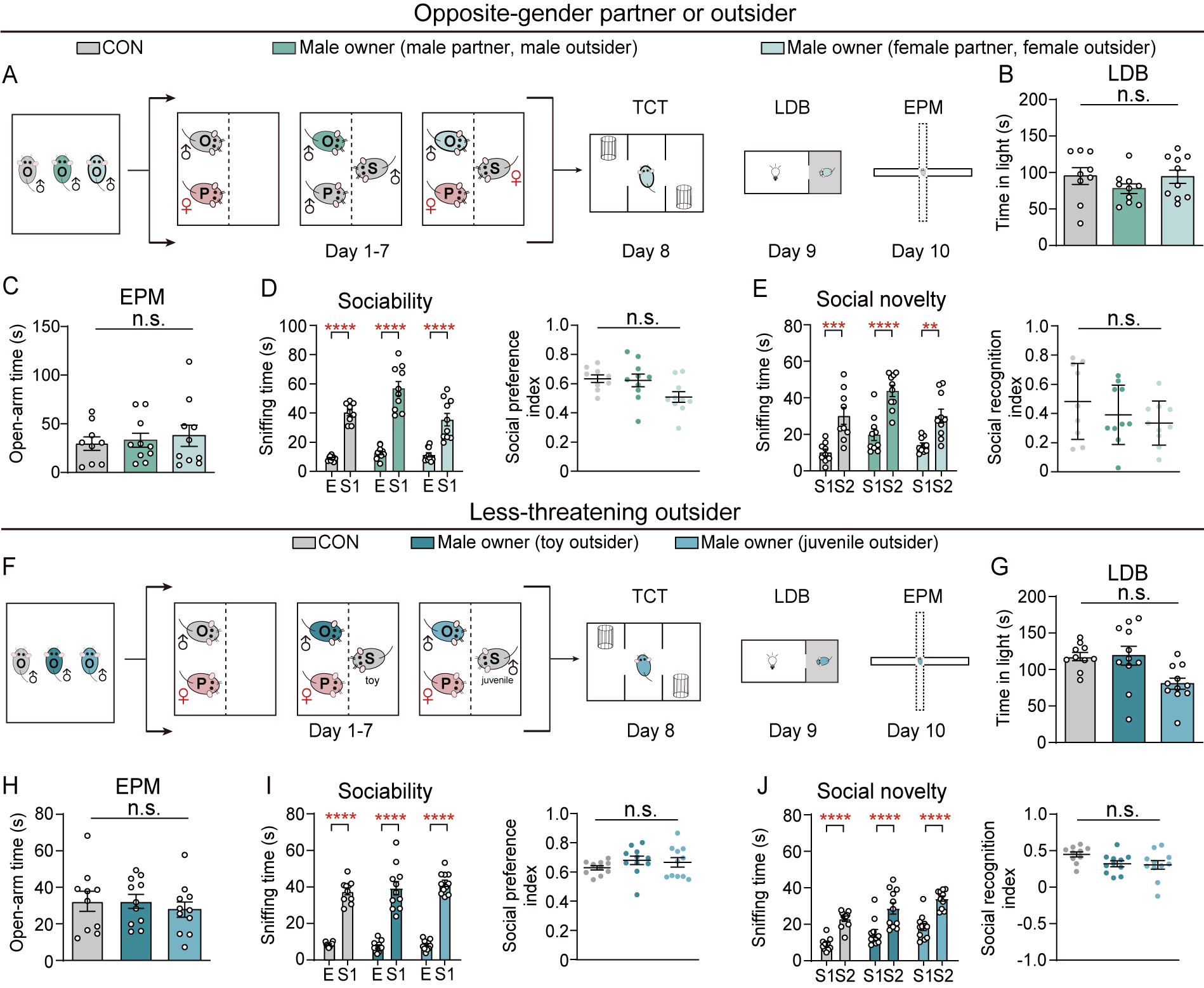
**

**Fig. S2. Female partner and adult male outsider are required for CCS model.**

(A) Paradigm for modified CCS model with sex of partner or outsider changed. (B-E) Quantification of time spent in light chamber in the LDB (B), time spent in open arms in the EPM (C), and sniffing time and social indexes in TCT (D, E). (F) Experimental procedure for behavioral tests of male owner mice exposed to different outsiders. Toy and juvenile mice (~4 weeks old) represent outsiders of less threat to owner mice. (G-J) Quantification of time spent in light chamber in the LDB (G), time spent in open arms in the EPM (H), and sniffing time and social indices in TCT (I, J). Values are means ± SEM. ***P* < 0.01, ****P* < 0.001, *****P* < 0.0001, n.s., no significance (see table S1 for statistics and n numbers).

**
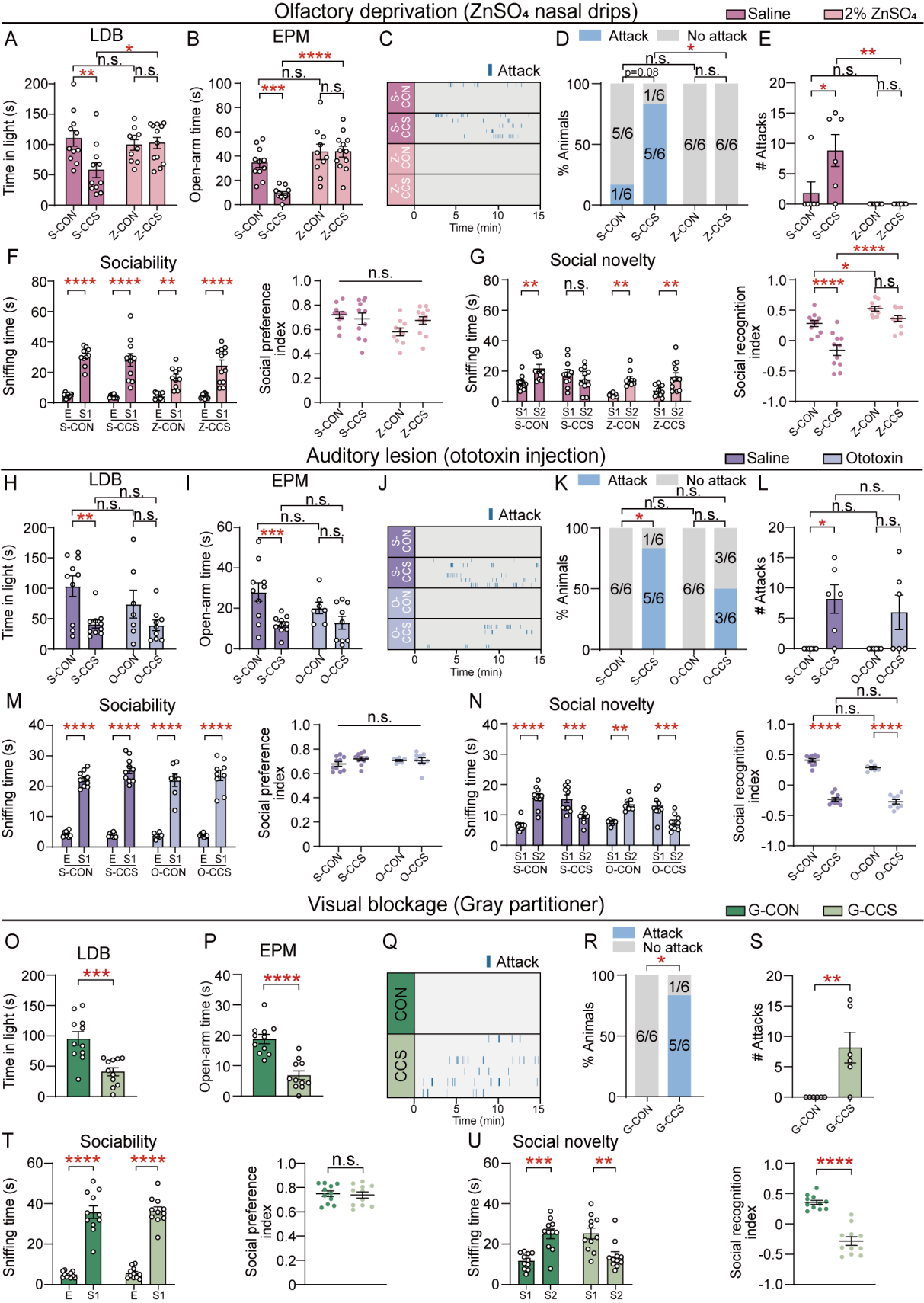
**

**Fig. S3. Olfactory deprivation, but not auditory lesion or visual blockage, prevents anxiety-like and aggressive behaviors.**

(A-G) Time spent in light chamber in the LDB (A), time spent in open arms in the EPM (B), raster plots of attacks (C), percentage of owners showing attacks (D), number of outsider-directed attacks (E), and sniffing time along with indices for sociability (F) and social novelty (G) in TCT of CON and CCS male mice treated with intra-nasal drips of 20 μl of saline or ZnSO4 (2%) one day before modeling. S-CON: saline-treated CON mice; S-CCS: saline-treated CCS mice; Z-CON: ZnSO_4_-treated CON mice; Z-CCS: ZnSO_4_-treated CCS mice. (H-N) Time spent in light chamber in the LDB (H), time spent in open arms in the EPM (I), raster plots of attacks (J), percentage of owners showing attacks (K), number of outsider-directed attacks (L), and sniffing time along with indices for sociability (M) and social novelty (N) in TCT of CON and CCS male mice treated with i.p. injection of saline or ototoxin 3 days before modeling. S-CON: saline-treated CON mice; S-CCS: saline-treated CCS mice; O-CON: ototoxin-treated CON mice; O-CCS: ototoxin-treated CCS mice. (O-U) Time spent in light chamber in the LDB (O), time spent in open arms in the EPM (P), raster plots of attacks (Q), percentage of owners showing attacks (R), number of outsider-directed attacks (S), and sniffing time along with indices for sociability (T) and social novelty (U) in TCT of CON and CCS male owners separated from outsiders by gray opaque partitions during modeling. G-CON: CON mice separated by gray opaque partitions; G-CCS: CCS mice separated by gray opaque partitions. Values are means ± SEM. except (D, K, R). **P* < 0.05, ***P* < 0.01, ****P* < 0.001, *****P* < 0.0001, n.s., no significance (see table S1 for statistics and n numbers).

**
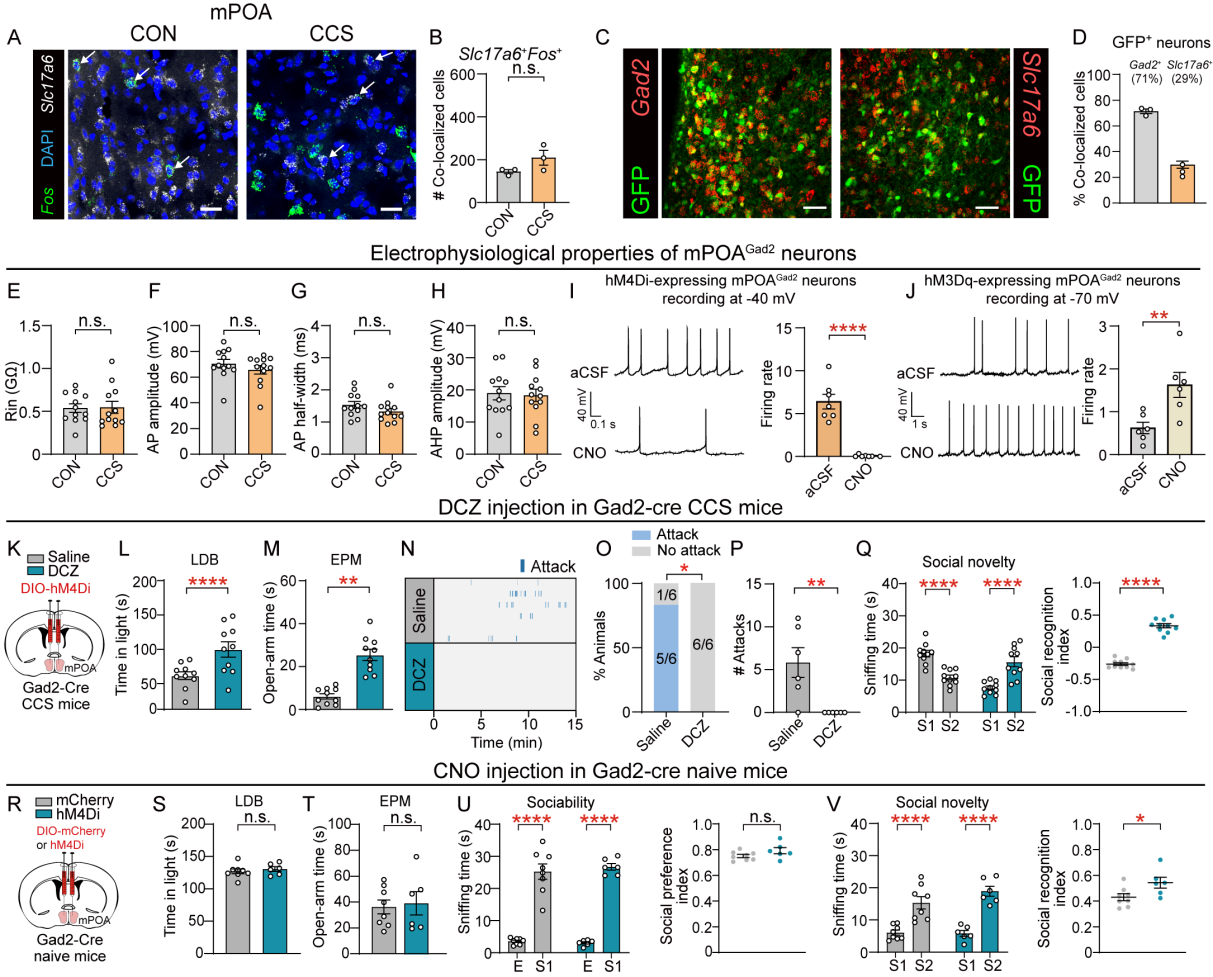
**

**Fig. S4. mPOA^Gad2^ neurons mediate anxiety-like and aggressive behaviors induced by CCS.**

(A, B) Representative images (A) and quantification (B) of cells co-expressing *Slc17a6* and *Fos* in the mPOA of CON and CCS mice. White arrows indicate *Fos^+^* neurons expressing *Slc17a6.* Scale bar, 20 μm. (C, D) Representative images (C) and quantification (D) of the colocalization between GFP and *Gad2* or *Slc17a6* (C) in the mPOA of *C57* mice with intra-mPOA infection of GAD2-GFP virus. Scale bar, 50 μm. (E-H) Quantification of input resistance (E), AP amplitude (F), AP half-width (G), and AHP amplitude (H) of mPOA^Gad2^ neurons in CON and CCS mice. (I) Firing rate of hM4Di-expressing mPOA^Gad2^ neurons recorded at -40 mV before and after CNO perfusion. (J) Firing rate of hM3Dq-expressing mPOA^Gad2^ neurons recorded at -70 mV before and after CNO perfusion. (K) hM4Di-expressing Gad2-Cre mice with CCS exposure received acute i.p. injection of saline or DCZ (0.1 mg/kg) 30 min before each test. (L-Q) Time spent in light chamber in the LDB (L), time spent in open arms in the EPM (M), raster plots of attacks (N), percentage of owners showing attacks (O), number of outsider-directed attacks (P), and sniffing time along with index for social novelty (Q) in TCT of DCZ-treated hM4Di-expressing Gad2-Cre CCS mice. (R) Paradigm for virus injection sites in Gad2-Cre naïve male mice. mCherry- or hM4Di-expressing mice received acute i.p. injections of CNO 30 min before each test. (S-V) Time spent in light chamber in the LDB (S), time spent in open arms in the EPM (T), and sniffing time along with indices for sociability (U) and social novelty (V) in TCT of CNO-treated hM4Di-expressing Gad2-Cre naïve male mice. Values are means ± SEM. **P* < 0.05, ***P* < 0.01, *****P* < 0.0001, n.s., no significance (see table S1 for statistics and n numbers).


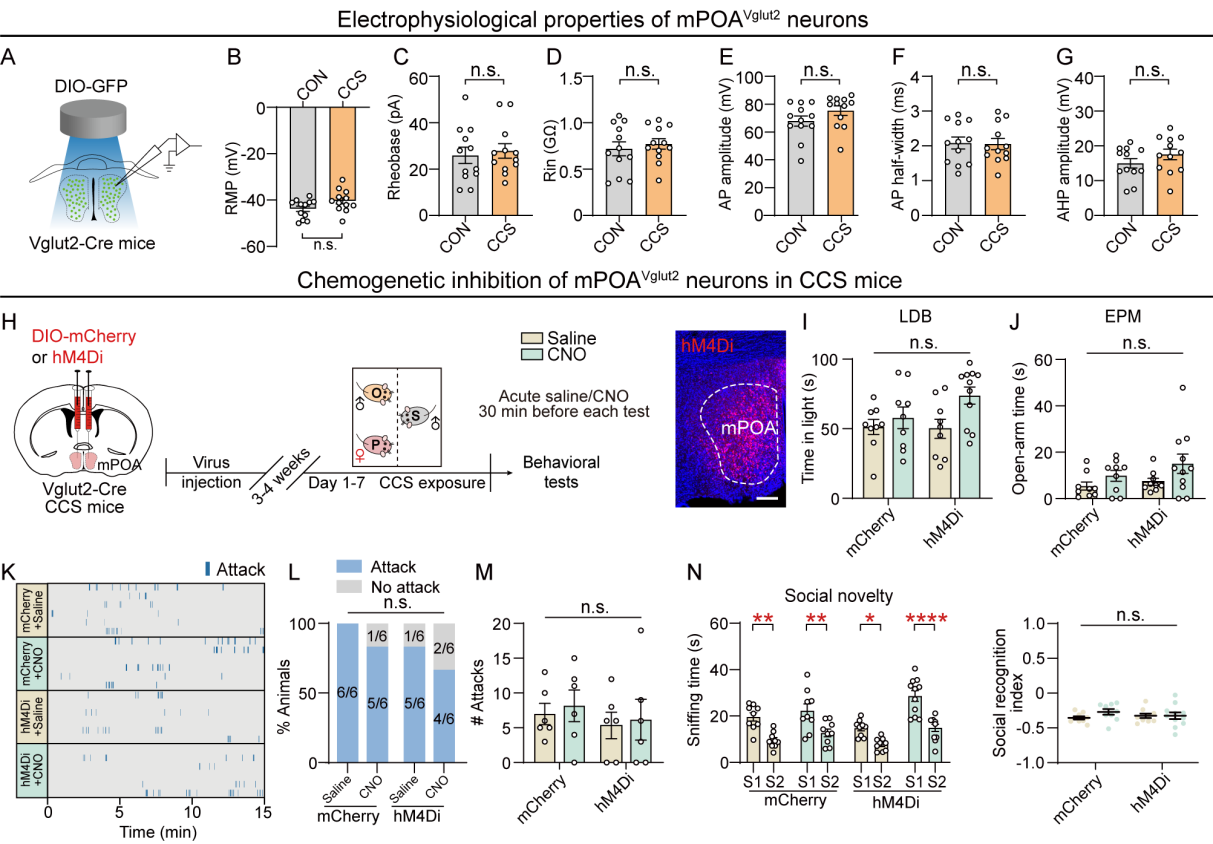


**Fig. S5. CCS exposure does not affect activity and excitability of the mPOA^Vglut2^ neurons.**

1. Schematic of electrophysiological recordings on mPOA^Vglut2^ neurons in CON and CCS mice. The mPOA^Vglut2^ neurons in male Vglut2-Cre owners were labeled by DIO-GFP injection in Vglut2-Cre mice. (B-G) Quantification of RMP (B), rheobase (C), and input resistance (D), AP amplitude (E), AP half-width (F), and AHP amplitude (G) of mPOA^Vglut2^ cells in CON and CCS mice. (H) Left, experimental procedure for testing behaviors of CCS mice with chemogenetic inhibition in mPOA^Vglut2^ neurons. Right, representative fluorescence image showing viral infection in the mPOA. Scale bar, 200 μm. (I) Quantification of time spent in light chamber in the LDB by viral-infected mice treated with saline or CNO. (J) Quantification of time spent in open arms in the EPM. (K) Raster plots showing outsider-directed attacks. (L) Percentage of CCS mice showing attacks. (M) Total number of outsider-directed attacks launched by CCS mice. (N) Quantification of sniffing time for social novelty and social recognition index in TCT. Values are means ± SEM. **P* < 0.05, ***P* < 0.01, *****P* < 0.0001, n.s., no significance (see table S1 for statistics and n numbers).


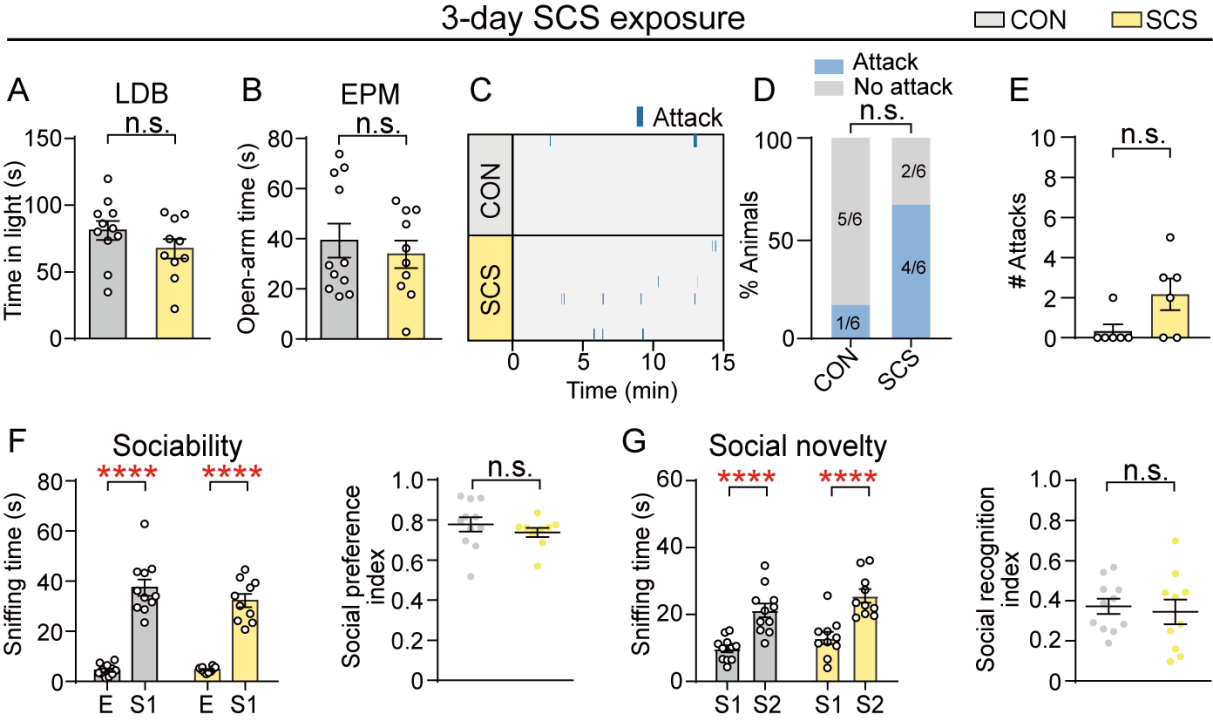


**Fig. S6. 3-day SCS exposure does not induce anxiety-like and aggressive behaviors in male owners.**

(A) Quantification of time spent in light chamber in the LDB by male owner mice subject to 3-day CON or SCS paradigm. (B) Quantification of time spent in open arms in the EPM. (C) Raster plots of attack launched against outsiders. (D) Percentage of SCS owners showing attacks. (E) Total number of outsider-directed attack launched by SCS mice. (F, G) Quantification of sniffing time and social indexes in TCT. Values are means ± SEM. except (D) . **P* < 0.05, ***P* < 0.01, *****P* < 0.0001, n.s., no significance (see table S1 for statistics and n numbers).

**
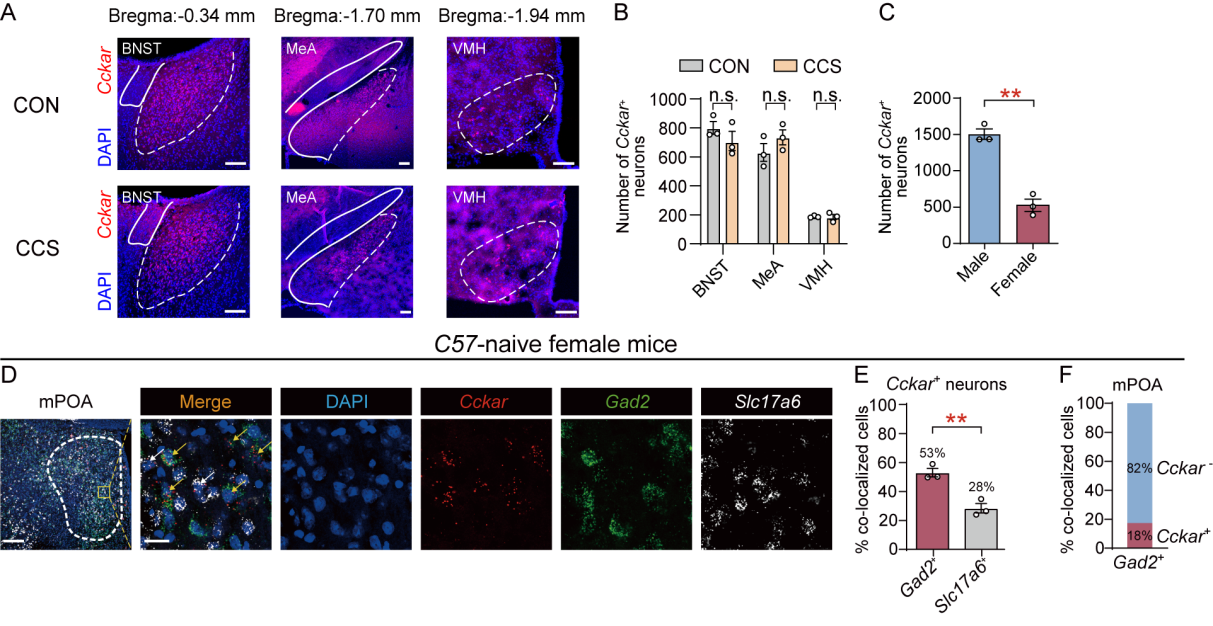
**

**Fig. S7. Sexually dimorphic expression of *Cckar* in the mPOA.**

(A, B) Representative images (A) and quantification (B) of *Cckar^+^* neurons in the BNST, MEA, and VMH for in CON and CCS mice. Scale bar, 100 μm. (C) Total number of *Cckar*^+^ neurons in naïve male and female mice. (D) Representative RNAscope images of *Cckar*, *Gad2*, and *Slc17a6* mRNA in mPOA of *C57*-naïve female mice. Yellow arrows indicate *Cckar^+^* neurons expressing *Gad2*; white arrows indicate *Cckar^+^* neurons expressing *Slc17a6*. (E, F) Percentage of *Cckar^+^* neurons expressing *Gad2* or *Slc17a6* (E) and proportion of *Cckar^+^* neurons cells in mPOA^Gad2^ neurons in *C57*-naïve female mice (F). Values are means ± SEM. ***P* < 0.01 (see table S1 for statistics and n numbers).

**
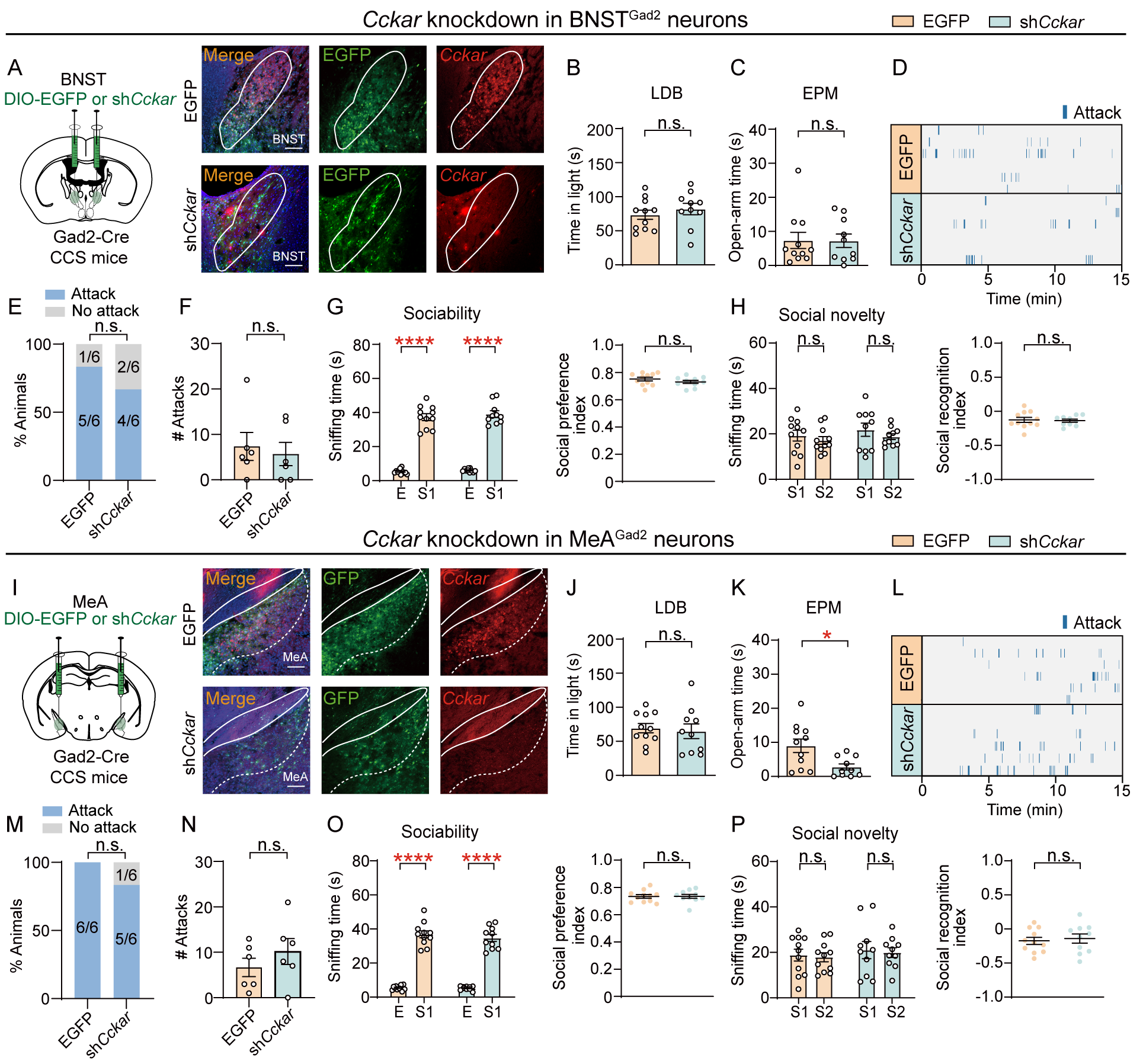
**

**Fig. S8. *Cckar* knockdown in BNST and MeA GABAergic neurons does not improve anxiety-like and aggressive behaviors in CCS mice.**

(A and I) Left, schematic of viral injection for conditional knockdown of *Cckar* in BNST^Gad2^ neurons. Right, representative fluorescence images showing expression of viral EGFP protein and *Cckar* mRNA in BNST (A) or MeA (I) of Gad2-Cre CCS mice injected with control (EGFP) or knockdown (sh*Cckar*) virus. Scale bar, 100 μm. (B and J) Quantification of time spent in light chamber in the LDB test. (C and K) Quantification of time spent in open arms in the EPM test. (D and L) Raster plots showing outsider-directed attacks from EGFP and sh*Cckar*-expressing CCS mice. (E and M) Percentage of EGFP and sh*Cckar*-expressing CCS mice showing attacks. (F and N) Total number of outsider-directed attacks launched by EGFP and sh*Cckar*-expressing CCS mice. (G and O) Quantification of sniffing time for sociability and social preference index in TCT. (H and P) Quantification of sniffing time for social novelty and social recognition index in TCT. Values are means ± SEM. except (E) and (M). **P* < 0.05, *****P* < 0.0001, n.s., no significance (see Table S1 for statistical analyses and n numbers).

**
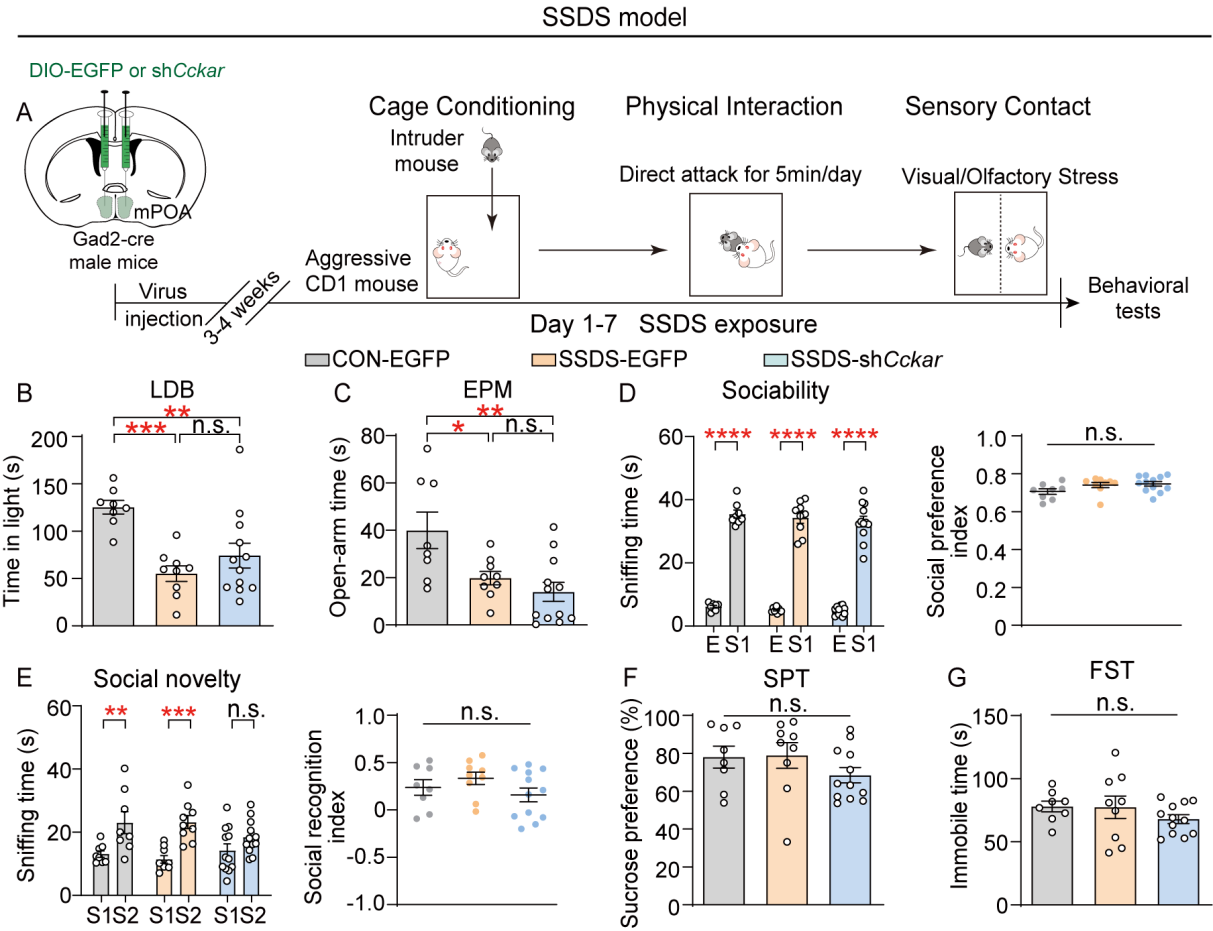
**

**Fig. S9. *Cckar* knockdown in mPOA^Gad2^ neurons neither rescues anxiety-like behaviors nor influences social or depression-like behaviors in SSDS mice.**

(A) Experimental procedure for behavioral tests of *C57* male mice with mPOA^Gad2^ neuron-specific *Cckar* knockdown after a 7-day SSDS paradigm. C-EGFP: mice with EGFP expression but no SSDS exposure. SSDS-EGFP: mice with EGFP expression and SSDS exposure. SSDS-sh*Cckar*: mice with mPOA^Gad2^ neuron-specific *Cckar* knockdown and SSDS exposure. (B) Time spent in light chamber in the LDB. (C) Time spent in open arms in the EPM. (D, E) Quantification of sniffing time and social indexes in TCT. (F, G) Quantification of depression-like behaviors including sucrose preference (F) and immobility time in FST (G). Values are means ± SEM. **P* < 0.05, ***P* < 0.01, ****P* < 0.001, *****P* < 0.0001, n.s., no significance (see table S1 for statistics and n numbers).


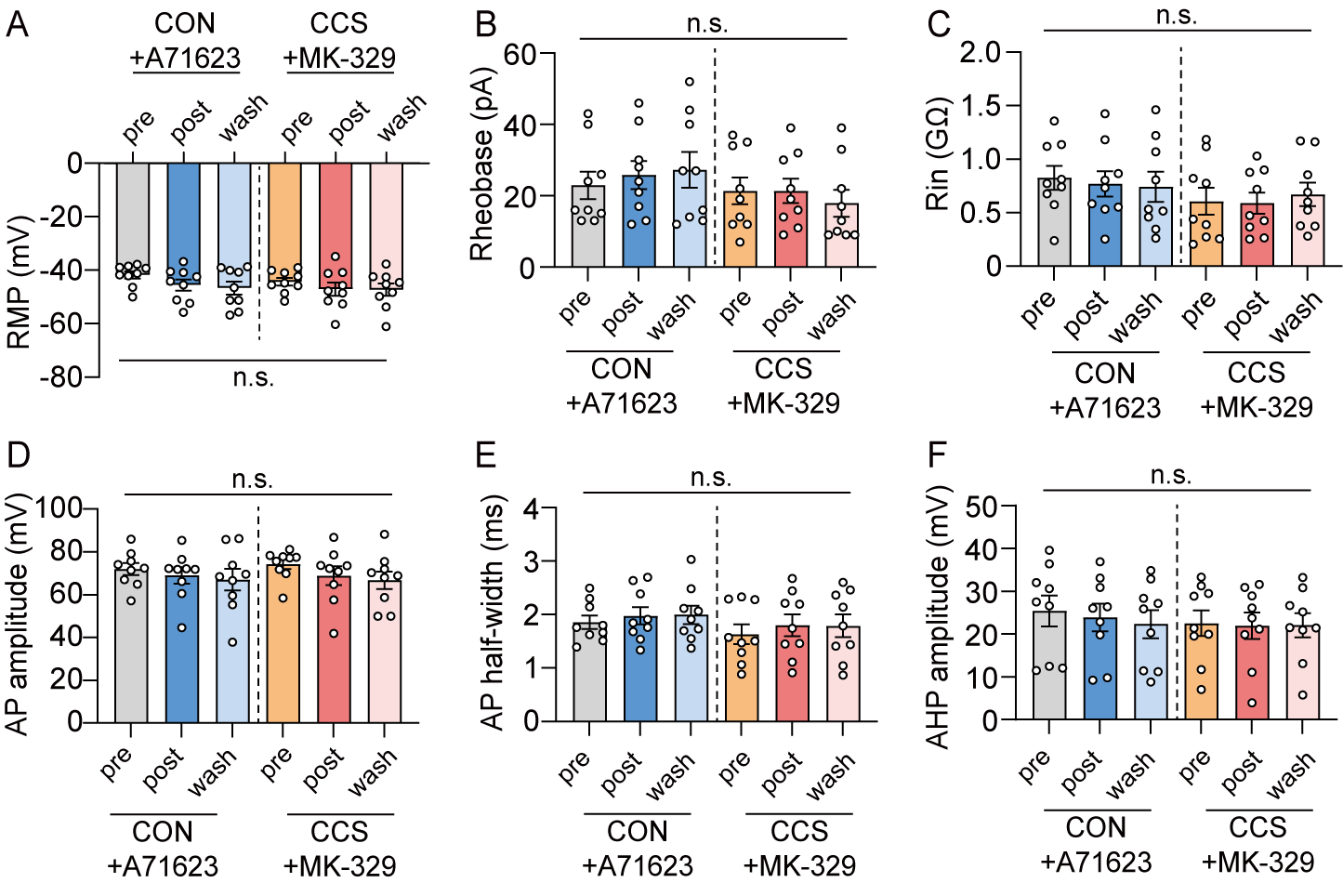


#### Fig. S10. CCKAR does not regulate excitability of mPOA^Vglut2^ neurons.

(A-F) Quantification of RMP (A), rheobase (B), and input resistance (C), AP amplitude (D), AP half-width (E), and AHP amplitude (F) of labeled mPOA^Vglut2^ neurons in CON and CCS male mice at stages of pre-/post-incubation and washout of CCKAR antagonist (MK-329) or agonist (A71623). Values are means ± SEM. n.s., no significance (see table S1 for statistics and n numbers).


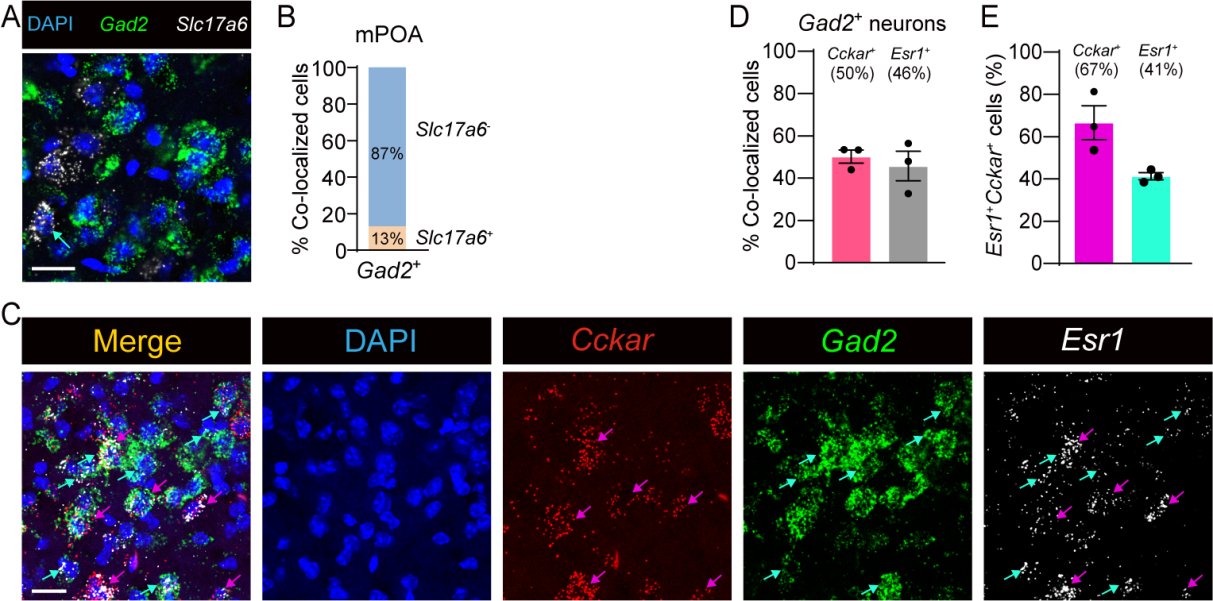


#### Fig. S11. mPOA^Gad2^ neurons partially co-localized with *Slc17a6* , *Cckar* and *Esr1*.

(A) Representative RNAscope images showing *Gad2*, and *Slc17a6* expression in the mPOA of naïve *C57* male mice related to Fig. 3E (scale bar, 20 μm). Green arrow indicates *Gad2^+^Slc17a6^+^* neurons. (B) Percentage of co-localization between *Gad2* and *Slc17a6*. (C) Representative RNAscope images showing *Cckar*, *Gad2*, and *Esr1* expression in the mPOA of naïve *C57* male mice (scale bar, 20 μm). Magenta arrows indicate *Cckar^+^* neurons expressing *Esr1*^+^; cyan arrows indicate *Gad2*^+^ neurons expressing *Esr1*^+^. (D) Percentage of *Gad2*^+^ neurons expressing *Cckar* or *Esr1*. (E) Proportion of *Esr1*^+^*Cckar*^+^ cells in *Cckar*^+^ or *Esr1*^+^ neurons. Values are means ± SEM. n.s., no significance (see table S1 for statistics and n numbers).
