# Supplementary material for: Medial preoptic CCKAR mediates anxiety and aggression induced by chronic emotional stress in male mice
Source: Natl Sci Rev. 2025 May 21;12(10):nwaf152. doi: 10.1093/nsr/nwaf152 (PMC12418936; doi:10.1093/nsr/nwaf152)
Supplement: nwaf152_Supplemental_Files [file nwaf152_supplemental_files.zip › Supplementary Files.docx]

## METHOD DETAILS

## Viruses

Chemogenetic viral vectors, including pAAV2/9-hsyn-DIO-mCherry (6.9 × 10^12^), pAAV2/9- hsyn-DIO-hM4Di-mCherry (8 × 10^12^), and pAAV2/9-hsyn-DIO-hM3Dq-mCherry (8.5 × 10^12^) were purchased from OBIO. The target sequence of *Cckar* short hairpin RNA (shRNA) was 5’-CGTGAGTGTTTCCACCTTCAA-3’. *Cckar* knockdown and sham viruses, including pAAV2/9-CMV-DIO-EGFP-miR30shRNA (*Cckar*)-WPRE (1.30 × 10^13^), pAAV2/9-CMV-DIO-EGFP-miR30shRNA (NC)-WPRE (1.27 × 10^13^); *Cckar*-overexpression and sham viruses, including pcAAV2/9-CMV-DIO-*Cckar*-3xFLAG-P2A-EGFP-WPRE (5.77 × 10^12^), and pcAAV2/9-CMV-DIO-EGFP-WPRE (1.70 × 10^13^) were obtained from OBIO. *Gad2*^+^ neurons were labeled with rAAV/9-mGAD65-EGFP (1.35 × 10^12^, purchased from Brain Case) for slice recordings. All virus was allowed 3-4 weeks for completely expression before any tests.

## Stereotaxic surgeries

Stereotaxic viral injection and cannula implantation were performed as described in previous research[1]. In brief, anesthetized mice were placed on a stereotaxic apparatus (RWD Life Science), with 60 nl of virus bilaterally injected into the target area using syringe pumps (KD Scientific) at 30 nl/min. The needle was left in place for at least 10 min before withdrawal. Stereotaxic coordinates were based on the Paxinos and Franklin Atlas (4th edition) as follows: mPOA (AP, +0.15 mm; ML, ±0.35 mm; DV, −5.00 mm), BNST (AP, −0.2 mm; ML, ±0.8 mm; DV, −4.00 mm), and MeA (AP, −1.70 mm; ML, ±2.23 mm; DV, −4.92 mm). For pharmacological experiments, a bilateral cannula (RWD Life Science) was implanted above the mPOA (AP, +0.15 mm; ML, ±0.35 mm; DV, −4.9 mm) for infusion of the CCKAR antagonist MK-329 (300 μM, MCE) and CCKAR agonist A71623 (200 μM, MCE). Only mice with correct cannula placement and viral expression were used for further analysis.

## Model

### Chronic conspecific outsider stress (CCS) model

In the male-specific 7-day CCS model (Fig. 1), group-housed healthy male mice of similar weight were randomly assigned into either control (CON) or CCS groups. Each cage was divided into two halves by a transparent, perforated, and detachable partition, which prevented physical contact but permitted sensory interactions between mice on both sides. Both CCS and CON groups contained a male owner mouse and a female partner in one compartment while CCS group contained a peer and stranger *C57* male outsider in the adjacent compartment and CON group included an empty adjacent compartment. To minimize the influence by pregnancy, virgin females or females that had been separated from males for more than 2 weeks were randomly assigned as partners. Any data from females showing signs of pregnancy during the modeling period were excluded. All the mice were placed into a novel fresh cage at the same time.

In the modified female CCS paradigm (Fig. S1), a female owner was paired with a male partner on one side of the cage, while a female or male outsider was housed on the other side. Substitutions of male CCS paradigm involving the replacement of partners, and outsiders with opposite-sex mice, inanimate toy mice (devoid of smell, sound, or motion), or juvenile mice (~4 weeks old) were conducted as specified in the text (Fig. S2). For sensory blockage (Fig. S3), 20 μl of 2% ZnSO_4_ bilaterally dripped into the nasal cavities was used to inhibit olfactory perception [2]. Intraperitoneal injection of kanamycin (1,000 mg/kg, MCE) followed 30-45 min later by a single dose of furosemide (400 mg/kg, MCE) was applied for hearing loss 3 days before modeling [3]. A gray opaque partition was used to obscure vision.

In the 3-day subthreshold conspecific stress (SCS) model (Fig. S5), the procedure mirrored the standard 7-day CCS paradigm but spanned only 3 consecutive days.

### Subthreshold social defeat stress (SSDS)

The 7-day SSDS paradigm used in this study has been modified as described previously[4]. In brief, aggressive male CD1 mice were screened before modeling. In the SSDS group, over a span of 7 consecutive days, each experimental mouse, acting as an intruder, was exposed to an unfamiliar resident CD1 mouse to experience social defeat for 5 min. Subsequently, the defeated mice were physically isolated from the CD1 mice by a partition positioned in the center of the enclosure, permitting sensory interaction for 24 h. Control mice (CON) were housed with *C57*-naïve mice in the same two-chamber cage, receiving no physical or sensory contact with CD1 mice. Following the 7-day stress regimen, the mice were housed individually for 24 h before being evaluated through behavioral assessments.

## Behavioral tests

Behavioral assessments included aggressive behavior recording and sequential three-chamber social preference test (TCT), light/dark box test (LDB) and elevated plus-maze test (EPM). When required, sucrose preference test (SPT) and forced swim test (FST) were finally conducted to evaluate depression-related behaviors. All tests were separated by a minimum 24-h interval.

### Aggressive behavior recording

Aggressive behaviors were video recorded by Logitech cameras mounted above the cages for 15 minutes using Bandicam software. These recordings were conducted with a separate cohort of mice or 24 hours post the completion of other behavioral assessments. To facilitate subsequent manual tagging of aggressive incidents in the open-source event-logging software BORIS [5], male residents and intruders were distinctly marked for identification. Aggression was characterized by a sequence of behaviors, including chasing, biting, lunging, and wrestling, initiated by the resident mice towards the outsiders. The number of attacks and corresponding raster plots were directly exported from BORIS for further analysis.

### LDB

The light-dark box apparatus featured two compartments separated by a partition with a door (5 cm × 5 cm × 5 cm). The light compartment (20 cm × 30 cm × 25 cm) was illuminated by a 100 W desk lamp, while the dark compartment (20 cm × 15 cm × 25 cm) was covered with a black cover. After habituation in the test room for 30 min, mice were placed in the dark compartment and allowed to freely explore the two compartments for 5 min. Time spent in the light compartment was manually recorded.

### EPM

The elevated plus-maze apparatus consisted of two open arms (30 cm × 5 cm) and two enclosed arms (30 cm × 5 cm × 15 cm) extending from a central intersecting platform (5 cm × 5 cm), positioned 50 cm above the ground. The mice were habituated to the test room for 30 min. Subsequently, each mouse was placed on the central platform facing one open arm and allowed to freely explore for 5 min. Locomotion was tracked for 5 min in real time using ANY-maze software. Conflict avoidance behaviors were evaluated based on time spent in the open arms.

### TCT

The three-chamber apparatus was divided into three 20-cm-wide compartments, with two wire mesh covers placed in the left/right compartments diagonally. The test comprised three stages, each lasting 5 min. In the first stage (habituation), an experimental mouse was placed in the center compartment and allowed to freely explore. Social mice were 3-4 week old of the same sex with experimental mice. In the second stage, a social mouse (same-sex, juvenile mouse) was placed under one of the wire mesh covers (Stranger 1, S1), while the opposite enclosure remained empty (E). In the third stage, a new social mouse was introduced into the previously empty enclosure (Stranger 2, S2). Mice were tracked using ANY-maze software and time spent sniffing S1 or S2 by the experimental mouse was manually recorded. Social preference index was calculated by: (S1 − E) × 100 / (S1 + E) and the social recognition index was calculated by: (S2− S1) × 100 / (S2 + S1).

### SPT

Single-housed mice were provided with a bottle of water and a bottle of 1% sucrose solution for 3 consecutive days, with bottle positions switched twice a day (09:00 and 18:00) to avoid side preference. The bottles were removed on the third day at 18:00 and returned the following morning of the fourth day after being weighed. Final weights of the bottles were measured at 2 h after the test started, with bottle positions switched in the middle. Sucrose preference was calculated as the amount of sucrose consumed divided by the total weight of liquid (water plus sucrose) consumed.

### FST

Mice were kept in a plexiglass container (30-cm deep, 46-cm tall, 20-cm diameter) filled with water (25 ± 1 °C) for 6 min. Immobility time of mice in the last 4 min was manually determined as an assessment of depression.

## Immunohistochemistry

Immunochemical analysis was performed as described in previous research[1]. Deeply anesthetized mice were perfused, and their brains were removed, post-fixed, and dehydrated. Frozen sections (50μm) were sliced using a cryostat (Leica CM1950) and stored in 0.1 M phosphate-buffered saline (PBS) solution. The brain slices were then mounted on adhesion microscope slides with 100 μL of DAPI-Fluoromount-G (Southern Biotech, Beijing, China). Images for injection sites were acquired using a slide scanner (Olympus VS120).

## RNAscope *in situ* hybridization

Fluorescence *in situ* hybridization for coronal brain sections (20 μm) was conducted using an RNAscope Multiplex Fluorescent Reagent Kit v2 and corresponding probes, including RNAscope Probe-Mm-*Cckar* (313758-C1), RNAscope Probe-Mm-*Gad2* (439371-C2), RNAscope Probe-Mm-*Slc17a6* (319171-C4), RNAscope Probe-Mm-*Fos* (316921-C1), RNAscope Probe-Mm-*Esr1* (478201-C4), RNAscope Probe-Mm-*Gad2* (439371-C1), and RNAscope Probe-Mm-*Slc32a1* (319191-C2) (ACDbio), following the manufacturer’s recommended protocols. Well-validated RNAscope negative control probes were used as instructed. Images were captured using a confocal microscope (Olympus FV3000) and cells were counted manually using ImageJ.

## Corticosterone enzyme-linked immunosorbent assay (ELISA)

Blood samples were obtained via cardiac puncture into EDTA tubes, followed by centrifugation at 3 000 rpm for 10 min at 4 °C. Serum corticosterone levels were quantified using a corticosterone competitive ELISA kit (ThermoFisher) following the manufacturer’s instructions. The samples, diluted to 1:100, were assayed in triplicate and compared against a standard curve. The average of the triplicate measurements for each animal was determined using a Varioskan Flash microplate reader (ThermoFisher).

**Western blot**

Brains from anesthetized mice were collected, rinsed with ice-cold PBS, and sliced using a mouse brain matrix (RWD Life Science, Shenzhen, China). The mPOA tissues were harvested from the slices for immediate protein extraction or rapidly frozen in liquid nitrogen (stored at -80°C). The tissues were homogenized with ice-cold RIPA buffer (Byotime, #P0013B) supplemented with protease and phosphatase inhibitor cocktails (Thermofisher Scientific, #A32961) for 1 min, and then lysed on ice for 30 min. Supernatant was collected after 15 min 14000 rpm centrifugation at 4 °C. Total protein concentration in the lysates was determined using a BCA kit (ThermoFisher Scientific, #23227). The lysates were mixed with 5 × sample loading buffer (Genescript, #MB01015) and incubated at 70 °C for 10 min for protein denaturation. Protein samples (20 μg) were then loaded onto a 4-20 % precast SDS-polyacrylamide gel (Genescript, #M42010C), and electrophoresis was performed in 1 × MOPS running buffer (Genescript, #M00138) at 100 V for 1 h. The resulting protein bands were transferred onto methanol-activated PVDF membranes in 1×MOPS transfer buffer (Genescript, #M00139) at 100 V and 4°C for 1 h. The membranes were then blocked with 5% milk in Tris-buffered saline containing 0.1% Tween-20 (TBST) and incubated with diluted primary antibody (rabbit anti-CCKAR, LSBio, #C382513, 1:500; rabbit anti-ESR1, Abcam, #ab32063, 1:500; rabbit anti-CALCR, Abcam, #ab11042, 1:500; rabbit anti-AR, Abcam, #ab133273, 1:500; mouse anti-GAPDH, ThermoFisher Scientific, #MA5-15738, 1:2000) at 4 °C overnight. The membranes were then washed using TBST and incubated with diluted secondary antibody (anti-rat IgG-HRP, Cell signaling technology, #7071, 1:3000; anti-mouse IgG (H+L)-HRP, MultiSciences, #70-GAM0072, 1:4000) at room temperature for 1 h. Following another TBST wash, the membranes were developed using ECL solution (ThermoFisher Scientific, #34577) and imaged using the Bio-Rad ChemiDoc imaging system. Data were analyzed using ImageJ.

## *Ex* vivo electrophysiology

Coronal brain slices containing the mPOA (250 μm thickness) were cut in cold high-sucrose artificial cerebrospinal fluid (aCSF) containing (in mM): 2.5 KCl, 1.25 NaH_2_PO_4_, 26 NaHCO_3_, 10 glucose, 230 sucrose, 0.5 CaCl_2_·2H_2_O, 10 MgSO_4_·7H_2_O and 1.5 sodium pyruvate using a vibratome (VT1200s, Leica) and then transferred to continuously oxygenated recording aCSF containing (in mM): 126 NaCl, 2.5 KCl, 1.25 NaH_2_PO_4_, 26 NaHCO_3_, 10 glucose, 2 CaCl_2_·2H_2_O, and 2 MgSO_4_·7H_2_O at room temperature. Patch pipettes (4–6 MΩ; borosilicate glass) were pulled using a micropipette puller (Model P-97, Sutter Instrument) for whole-cell patch-clamp recordings. Signals were acquired using an Axon Instruments MultiClamp 700B amplifier (low-pass filtered at 2 kHz, digitized at 10 kHz, Molecular Devices) and a 1440A interface (Molecular Devices) with pClamp (Molecular Devices). Gad2-GFP-labeled cells were visualized using a 40× water-immersion lens on an upright microscope (Eclipse FN1, Nikon) and illuminated with a mercury lamp. If series resistance changed by >20% during recording, the data were excluded from further analysis.

To identify mPOA^Gad2^ neurons and mPOA^Vglut2^ neurons during patch, we used *C57* mice infecting Gad2-GFP-reporter and Vglut2-cre mice infecting DIO-EGFP, respectively. All mPOA^Gad2^ neurons were recorded under current clamp mode to examine intrinsic membrane excitability and AP-related parameters. Electrodes were backfilled with an internal solution containing (in mM): 150 mM potassium gluconate, 5 mM NaCl, 1 mM MgCl_2_·6H_2_O, 2 mM Mg-ATP, 0.5 mM Na_3_-GTP, 0.2 mM EGTA and 10 mM HEPES (pH 7.3). Resting membrane potential (RMP) was determined at a holding current of zero (I = 0 pA) and without a predicted/measured junction potential correction. Input resistance (R_in_) was calculated from the linear fit of the neuronal voltage response to square-shaped current injections (500 ms, 10-pA steps) from -100 to 0 pA. Rheobase (the minimum current required to induce an AP) was first roughly measured by applying a 10-pA depolarizing current step (500 ms in duration) and then accurately measured by decreasing the step to 1 pA. Analysis of AP-related parameters was completed using Clampfit and Excel software. All data were collected 2 min after obtaining a stable whole-cell configuration.

**Statistical analysis**

Behavioral assays, histological samples and electrophysiological recordings were analyzed blind to experimental conditions. The methodological comparison of all experimental data was done by GraphPad PRISM, which was listed in detail in the table S1. The number of replicates (n) is indicated in the figure legends. All statistical significance followed the conventions: *P <0.05, **P <0.01, ***P <0.001, ****P <0.0001. Error bars represent ± SEM.

# REFERENCES

1. Shen C-J, Zheng D, Li K-X *et al.* Cannabinoid CB1 receptors in the amygdalar cholecystokinin glutamatergic afferents to nucleus accumbens modulate depressive-like behavior. *Nat Med* 2019;**25**:337–49.

2. Langford DJ, Crager SE, Shehzad Z *et al.* Social modulation of pain as evidence for empathy in mice. *Science* 2006;**312**:1967–70.

3. Oesterle EC, Campbell S, Taylor RR *et al.* Sox2 and Jagged1 expression in normal and drug-damaged adult mouse inner ear. *JARO* 2008;**9**:65–89.

4. Golden SA, Covington HE, Berton O *et al.* A standardized protocol for repeated social defeat stress in mice. *Nat Protoc* 2011;**6**:1183–91.

5. Friard O, Gamba M. BORIS : a free, versatile open‐source event‐logging software for video/audio coding and live observations. Fitzjohn R (ed.). *Methods Ecol Evol* 2016;**7**:1325–30.
